# Placental efflux transporters and antiseizure or antidepressant medication use impact birth weight in MoBa cohort

**DOI:** 10.1016/j.isci.2024.109285

**Published:** 2024-02-20

**Authors:** Marta H. Hernandez, Jacqueline M. Cohen, Karoline H. Skåra, Thea K. Grindstad, Yunsung Lee, Per Magnus, Pål R. Njølstad, Ole A. Andreassen, Elizabeth C. Corfield, Alexandra Havdahl, Espen Molden, Kari Furu, Maria C. Magnus, Alvaro Hernaez

**Affiliations:** 1 Centre for Fertility and Health, Norwegian Institute of Public Health, Oslo, Norway; 2 Blanquerna School of Health Sciences, University Ramon Llull, Barcelona, Spain; 3 Department of Chronic Diseases, Norwegian Institute of Public Health, Oslo, Norway; 4 Department of Community Medicine and Global Health, Institute of Health and Society, University of Oslo, Oslo, Norway; 5 Center for Diabetes Research, Department of Clinical Science, University of Bergen, Bergen, Norway; 6 Children and Youth Clinic, Haukeland University Hospital, Bergen, Norway; 7 Norwegian Centre for Mental Disorders Research, NORMENT, Division of Mental Health and Addiction, Oslo University Hospital, Oslo, Norway; 8 Institute of Clinical Medicine, University of Oslo, Oslo, Norway; 9 Center for Genetic Epidemiology and Mental Health, Norwegian Institute of Public Health, Oslo, Norway; 10 Nic Waals Institute, Lovisenberg Diakonale Hospital, Oslo, Norway; 11 PROMENTA Research Center, Department of Psychology, University of Oslo, Oslo, Norway; 12 MRC Integrative Epidemiology Unit, University of Bristol, Bristol, UK; 13 Center for Psychopharmacology, Diakonhjemmet Hospital, Oslo, Norway; 14 Section for Pharmacology and Pharmaceutical Department of Pharmacy, University of Oslo, Oslo, Norway

**Keywords:** Health informatics, Health sciences, Medical specialty, Medicine, Pharmacology, Psychiatry

## Abstract

Low birth weight raises neonatal risks and lifelong health issues and is linked to maternal medication use during pregnancy. We examined data from the Norwegian Mother, Father, and Child Cohort Study and the Medical Birth Registry of Norway, including 69,828 offspring with genotype data and 81,189 with maternal genotype data. We identified genetic risk variants in placental efflux transporters, calculated genetic scores based on alleles related to transporter activity, and assessed their interaction with prenatal use of antiseizure or antidepressant medication on offspring birth weight. Our study uncovered possible genetic variants in both offspring (rs3740066) and mothers (rs10248420; rs2235015) in placental efflux transporters (MRP2-*ABCC2* and MDR1-*ABCB1*) that modulated the association between prenatal exposure to antiseizure medication and low birth weight in the offspring. Antidepressant exposure was associated with low birth weight, but there were no gene-drug interactions. The interplay between MRP2-*ABCC2* and MDR1-*ABCB1* variants and antiseizure medication may impact neonatal birth weight.

## Highlights

• Prenatal antiseizure medication use impacts low birth weight depending on gene variants
• These are found in the *ABCC2* gene in the offspring and the *ABCB1* gene in mothers
• No gene-drug interaction for maternal antidepressant use on birth weight was found
• More studies are needed to guarantee the safe use of drugs during pregnancy

## Graphical abstract

**Figure:**
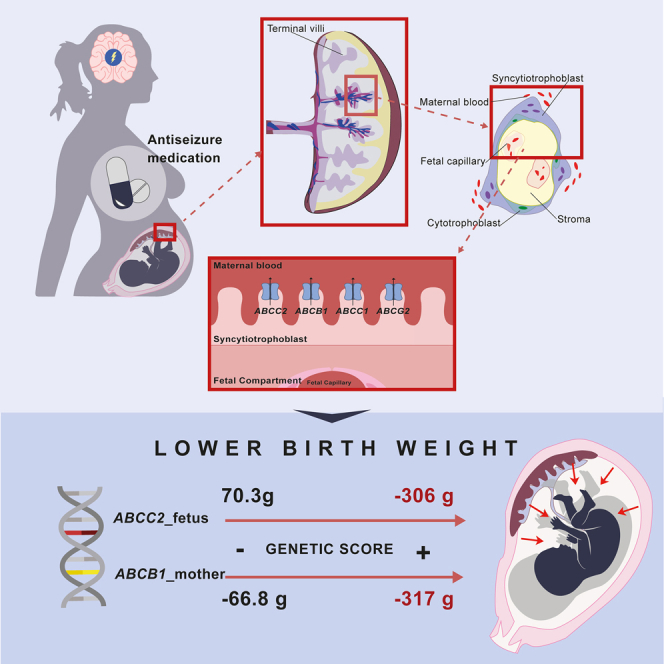

## Introduction

The use of medication during pregnancy must carefully balance benefits to the mother and potential harm to the offspring.^1^ Centrally acting drugs (including antiseizure medications [mainly lamotrigine, 0.3% of pregnancies] and antidepressants [1.5% of pregnancies]) have been increasingly used during pregnancy.^2^^,^^3^ However, intrauterine exposure to these medications may be associated with adverse pregnancy outcomes such as impaired fetal growth and reduced birth weight in offspring.^4^^,^^5^^,^^6^ Furthermore, these conditions are linked to a multitude of morbidities, resulting in both immediate and long-term adverse consequences for newborns, their families, and society.^7^ The placenta plays an essential role in substance exchange between mother and fetus (e.g., nutrients, metabolic by-products). Small, non-polar, lipophilic xenobiotics such as antiseizure medications and antidepressants can pass through the placenta via passive diffusion.^8^ However, several active proteins can efflux these compounds from the fetus back to the mother.^9^ Active efflux proteins from the adenosine triphosphate-binding cassette superfamily (such as P-glycoprotein, breast cancer resistance protein, and others multidrug resistance proteins) are involved in the transplacental passage of drugs, particularly affecting the passage of centrally acting drugs and their metabolites.^10^^,^^11^ Several antiseizure medications and antidepressants are substrates, to varying degrees, for these transporters,^12^^,^^13^^,^^14^ and transport gene expression can also be induced by antiseizure medications. Efflux transporters may therefore have a foetoprotective effect by lowering the concentrations of potentially toxic drugs on the fetal side. Genetic variants in the transporters may modify their activity and could be located in the offspring or maternal genotype.^15^^,^^16^ Little is known about the role of genetic variants in placental efflux transporters on the modulation of the association of antiseizure medications and antidepressants during pregnancy with offspring outcomes such as low birth weight.

Our aim was to explore whether there is an interaction between the use of antiseizure medications or antidepressants during pregnancy and genetic variants related to placental efflux transporters (in P-glycoprotein, breast cancer resistance protein, and other multidrug resistance proteins) and offspring birth weight.

## Results

### Study population

**Figure 1:**
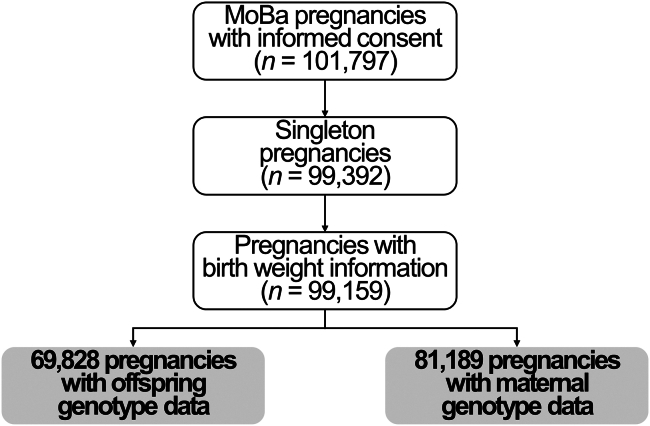
Flow chart of study participants

In our study, we included 69,828 singleton pregnancies with offspring genotype information and offspring birth weight and 81,189 singleton pregnancies with maternal genotype data and offspring birth weight (Figure 1). The mean birth weight was 3,639 g (SD 522). A total of 174 children (0.25%) were exposed to maternal use of antiseizure drugs during pregnancy and 766 (1.10%) to antidepressants. Women using centrally acting drugs were younger (only for antiseizure medications), had lower educational attainment, and were more likely to have ever smoked (Table 1). In the group of non-exposed individuals, we identified 40 (0.06%) women with a diagnosis of seizures and no antiseizure medication. Regarding depression, there were 5,580 (8.08%) women with a diagnosis of depression and no medication. We found no cases of people using both types of medication in our population.

**Table 1:** Baseline characteristics of mothers with offspring genotype information

	Antiseizure medications		Antidepressants	
	Exposed (n *=* 174)	Unexposed (n = 69,654)	Exposed (n = 766)	Unexposed (n = 69,062)
Age at delivery (years), mean ± SD	29.1 ± 4.83	30.2 ± 4.52	30.2 ± 5.07	30.2 ± 4.51
Education years, mean ± SD	15.9 ± 3.64	17.1 ± 3.30	16.2 ± 3.63	17.2 ± 3.30

**Pre-pregnancy body mass index categories:**				

Underweight (≤18.5 kg/m^2^)	4 (2.37%)	1,987 (2.93%)	35 (4.69%)	1,956 (2.91%)
Normal weight (18.5–24.9 kg/m^2^)	108 (63.9%)	44,684 (65.8%)	451 (60.4%)	44,341 (65.9%)
Overweight (25.0–29.9 kg/m^2^)	35 (20.7%)	14,872 (21.9%)	173 (23.2%)	14,734 (21.9%)
Obesity, type I and II (30.0–39.9 kg/m^2^)	20 (11.8%)	5,925 (8.73%)	80 (10.7%)	5,865 (8.71%)
Morbid obesity (≥40.0 (kg/m^2^)	2 (1.18%)	414 (0.61%)	8 (1.07%)	408 (0.61%)
Having ever smoked, *n* (%)	107 (61.8%)	34,310 (50.0%)	510 (67.4%)	33,907 (49.9%)

**Previous number of deliveries:**				

0, *n* (%)	64 (36.8%)	23,589 (33.9%)	261 (34.1%)	23,343 (33.9%)
1, *n* (%)	42 (24.1%)	22,342 (32.1%)	223 (29.1%)	22,113 (32.1%)
2, *n* (%)	44 (25.3%)	13,697 (19.7%)	156 (20.4%)	13,547 (19.7%)
3, *n* (%)	15 (8.62%)	6,118 (8.78%)	72 (9.40%)	6,045 (8.77%)
≥4, *n* (%)	9 (5.17%)	3,908 (5.61%)	54 (7.05%)	3,851 (5.59%)
Offspring birth weight, mean ± SD	3,516 ± 603	3,639 ± 522	3,574 ± 512	3,640 ± 522

**Biological sex of the offspring:**				

Female, *n* (%)	84 (48.3%)	35,549 (51.0%)	366 (47.8%)	35,267 (51.1%)
Male, *n* (%)	90 (51.7%)	34,105 (49.0%)	400 (52.2%)	33,795 (48.9%)

### Search of genetic variants on placental transporters

The systematic search identified 26 genetic variants in the placental transporter genes associated with differences in antiseizure medications and/or antidepressant outcomes (Figure S1), of which 14 were independent and available in the MoBa genotype database (Table S2). Seven variants were found for MDR1-*ABCB1*, two for MRP1-*ABCC1,* three for MRP2-*ABCC2*, and two for BCRP-*ABCG2*. We used these genetic variants to calculate risk scores. The ranges of values for the four genetic scores calculated were: 0 to 11 (MDR1-*ABCB1*; we presented the stratified associations according to quartiles), 0 to 4 (MRP1-*ABCC1*), 2 to 6 (MRP2-*ABCC2*), and 0 to 4 (BCRP-*ABCG2*).

### Interaction between genetic variants of placental transporters and exposure to antiseizure medications during pregnancy on birth weight

**Figure 2:**
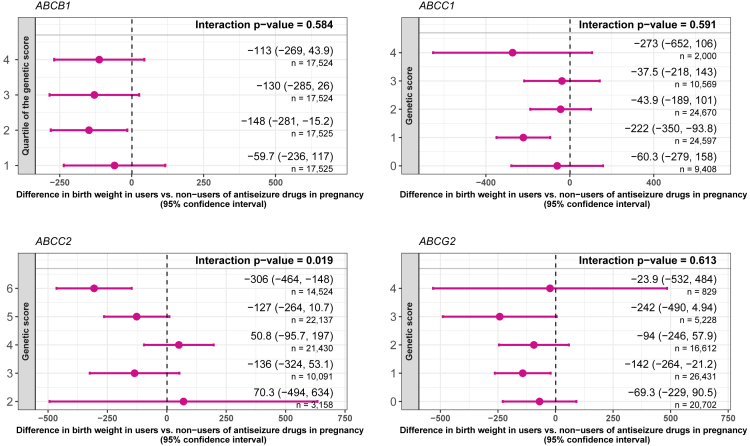
Differences in birth weight in offspring exposed to prenatal antiseizure medication (vs. not exposed) in groups defined by offspring genetic scores for efflux transporters

Antiseizure medication use during pregnancy was associated with a lower birth weight (−95.5 g, 95% confidence interval [CI] −190 to −0.78). Greater values of the MRP2-*ABCC2* genetic score in the offspring were associated with lower birth weight in the offspring of mothers who used antiseizure medications during pregnancy. The difference in birth weight between exposed vs. unexposed was 70.3 g (95% CI -494 to 634) in the lowest genetic score category (2 risk alleles) and −306 g (95% CI -361 to −31.8) in the highest (6 risk alleles) (p value for interaction = 0.019; Figure 2). The variant with the strongest evidence of an interaction in the sensitivity analyses of the MRP2-*ABCC2* genetic score was rs3740066 (p value for interaction = 0.023; Figure S2).

**Figure 3:**
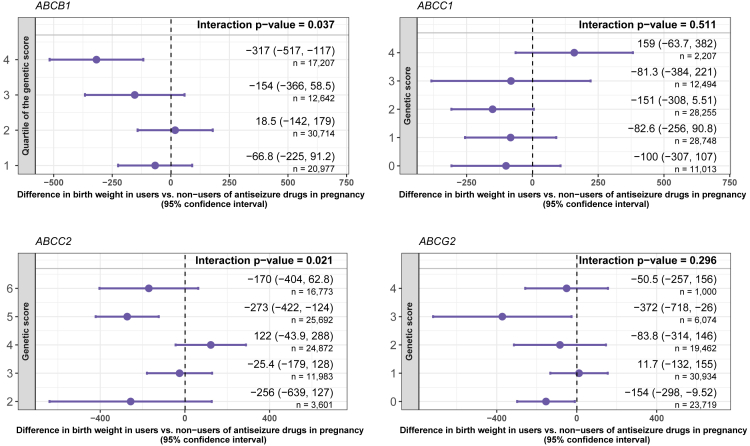
Differences in birth weight in offspring exposed to prenatal antiseizure medication (vs. not exposed) in groups defined by maternal genetic scores for efflux transporters

Greater values of the MDR1-*ABCB1* genetic score in the mother were also linked to lower birth weight among the offspring of mothers who used antiseizure medications during pregnancy. The difference in birth weight between exposed vs. unexposed was −66.8 g (95% CI -225 to 91.2) in the lowest genetic score category (first quartile) and −317 g (95% CI -517 to −117) in the highest (fourth quartile) (p value for interaction = 0.037; Figure 3). Variants rs10248420 and rs2235015 included in the MDR1-*ABCB1* genetic score had some evidence of an interaction in the sensitivity analyses (p values for interactions = 0.042 and 0.058, respectively; Figure S3).

### Interaction between genetic variants of placental transporters and exposure to antidepressants during pregnancy on birth weight

The use of antidepressant medication during pregnancy was associated with lower birth weight (−60.5 g, 95% CI -97.6 to −23.3). However, we observed no interactions between prenatal use of antidepressants and any genetic score of placental efflux transporters (in offspring or mothers) and birth weight in the offspring (Figures S4 and S5).

## Discussion

In this large population-based study, involving offspring of mothers who used antiseizure medication during pregnancy, a high genetic score for MRP2-*ABCC2* in the offspring and MDR1-*ABCB1* in the mother, suggesting a reduced performance of the efflux transporter, was associated with lower birth weight in the offspring MDR1-*ABCB1* and MRP2-*ABCC2* are efflux transporters located in the apical membrane of the trophoblast (of fetal origin on the maternal side), and their function is to return xenobiotics to maternal circulation.^17^^,^^18^ Thus, it seems plausible that genetic variants linked to lower transporter activity are associated with higher fetal exposure and greater toxicity of antiseizure medications. In addition, the main variant in the interaction between the MRP2-*ABCC2* genetic score and prenatal antiseizure medication use (rs3740066) has been previously linked to a poorer response to antiseizure medication^19^ and a higher risk of other adverse events during pregnancy (intrahepatic cholestasis of pregnancy).^20^ The differences in the interactions found within genetic variants in the offspring (MRP2-*ABCC2*) and maternal genome (MDR1-*ABCB1*) can be explained by physiological changes in the placenta structure (fetal origin). Early in pregnancy, the separation between the maternal circulation and the fetal circulation by the trophoblast villous barrier is 50–100 μm (second month). It becomes progressively thinner as the pregnancy proceeds (only 4–5 μm at term).^21^ In addition, the total placenta surface area increases from about 5 m^2^ at 28 weeks of gestation to 12 m^2^ at term. Thus, the passive diffusion of medications increases with gestational age, which means that medication can reach the fetal circulation more easily later in pregnancy.^21^ At the same time, the expression of MRP2-*ABCC2* increases with advancing gestational age, whereas the expression of MDR1-*ABCB1* declines.^22^^,^^23^ As a consequence of all these phenomena, the presence of risk alleles for MRP2-*ABCC2* may play a more significant role in determining the offspring’s exposure. However, an alternative hypothesis could also account for our findings. A higher fetal exposure to medication resulting from reduced efflux transporter activity might lead to lower medication levels in the mother and poorer disease control, which could increase the risk of low birth weight in the offspring by other mechanisms. For example, stress situations such as epilepsy and/or the use of antiseizure medication may modulate the expression and the function of efflux transporters,^13^ which, in turn, may also impact the exposure of the fetus to other potentially toxic xenobiotics.

Interactions with antidepressants. First, the association between exposure to antidepressants during pregnancy and birth weight is of a smaller magnitude in our data, which can make the search for gene-drug interactions more challenging. Second, the accuracy of our data regarding the usage of antidepressants may be less precise, as women may have varying criteria for identifying such treatments. Thirdly, 8% of the women in the group not exposed to antidepressants report presenting depression (contrarily to antiseizure medication), which could bias the inter-group comparison if some aspect of the pathology may impact the association between genetic variants of the efflux transporter and low birth weight in the offspring. Fourthly, antiseizure medications and epilepsy itself both may play a role in inducing these efflux transporters through alternative mechanisms. Finally, a larger proportion of women may discontinue the use of antidepressants during pregnancy compared to antiseizure medications.^24^

### Limitations of the study

Our study has some limitations. First, due to the unavailability of information on the specific drug utilized, we conducted our analysis based on aggregated drug groups. Although the main medications in each group show similar pharmacodynamic behavior and their usage during pregnancy reduces the number of medications under consideration,^3^ this may interfere with our findings because there may be variations in the medication groups’ interactions due to differences in transporter affinity or dose-dependent effects.^21^^,^^25^ Second, due to the lack of a genome-wide association study showing which genetic variants are associated with high or low activity of efflux transporters, we calculated risk scores using studies on genetic variants indirectly related to high or low activity (e.g., treatment resistance, treatment efficacy, presence of adverse effects). Third, there is considerable overlap between the lowest and highest scores of both gene variants for which we have described nominal interactions (*ABCC2* and *ABCB1*). These interactions are based on qualitative criteria, are only suggestive, and require replication in independent populations to confirm their validity. Fourth, some covariates, such as BMI, were measured before pregnancy, although it is the BMI values during pregnancy that could impact the risk of having a low-weight offspring. Thus, we assumed a strong correlation between pre-pregnancy and during-pregnancy measures. Finally, the characteristics of our participants (individuals of northern European ancestry with moderate-high socioeconomic status) limit our capacity to generalize our conclusions to other populations.

### Conclusions

In summary, genetic variants in MRP2-*ABCC2* and MDR1-*ABCB1* placental transporters may modulate the association between prenatal exposure to antiseizure medications and low birth weight in the offspring. To the best of our knowledge, this is the largest gene-drug interaction study on offspring birth weight to date, and it was performed in a well-characterized population with genome-wide genotype information. Our study highlights that interactions between efflux transporter genes and antiseizure medications during pregnancy may impact the birthweight of the offspring. Further research is needed to ensure the safe use of medications during pregnancy.

## STAR★Methods

### Key resources table

**Table:** 

REAGENT or RESOURCE	SOURCE	IDENTIFIER
**Software and algorithms**		

Code for analyses	R Software, version 4.1.0	GitHub: https://github.com/alvarohernaez/Gene_psychoactivedrug_BW_MoBa/.
TSD (Tjeneste for Sensitive Data)	University of Oslo	Access to datasets for replication should apply to datatilgang@fhi.no. Access to datasets requires approval from the Regional Committee for Medical and Health Research Ethics in Norway and an agreement with MoBa.

### Resource availability

#### Lead contact

Further information and requests for resources should be directed to and will be fulfilled by the lead contact, Marta H Hernandez (martahh1@blanquerna.url.edu).

#### Materials availability

This study did not generate new unique reagents.

#### Data and code availability

• Consent given by the participants does not open for storage of data on an individual level in repositories or journals. Researchers who want access to datasets for replication should apply to datatilgang@fhi.no. Access to datasets requires approval from the Regional Committee for Medical and Health Research Ethics in Norway and an agreement with MoBa.
• The code used to generate the study database and perform statistical analyses have been deposited at GitHub (GitHub: https://github.com/alvarohernaez/Gene_psychoactivedrug_BW_MoBa/) and is publicly available as of the date of publication.
• Any additional information required to reanalyse the data reported in this paper is available from the lead contact upon request.

### Experimental model and study participant details

#### Study participants

We included participants in the Norwegian Mother, Father, and Child Cohort Study (MoBa). MoBa is a prospective, population-based pregnancy cohort conducted by the Norwegian Institute of Public Health. Pregnant women and their partners were recruited across Norway between 1999-2008 at the time of routine ultrasound screening (∼17^th^ gestational week). The cohort includes approximately 114,000 children, 95,000 mothers, and 75,000 fathers.

This work used a subsample of offspring from singleton pregnancies with available information on genotype and birth weight (from version #12 of the quality-assured data files released on May 11, 2022). Genotype data was obtained from blood samples provided during pregnancy and at birth (mothers) and umbilical cord blood at birth (offspring).^26^ A total of 238,001 samples have been genotyped in 26 genotyping batches with varying selection criteria, genotyping platforms, and genotyping centres.^27^ The MoBaPsychGen pipeline was applied to this data to rigorously check quality at individual and gene variant levels.^27^ It used the Haplotype Reference Consortium release 1.1. for phasing and imputation and incorporated data from the Medical Birth Registry of Norway and MoBa questionnaires to identify personal details and relationships.^27^ The pipeline processed data from 207,569 unique individuals (90% of the study population) and over 6.98 million gene variants, and successfully identified familial relationships (first to third-degree relatives) within and across generations.

Our work is described according to the Strengthening the Reporting of Observational studies in Epidemiology guidelines (Table S1).^28^

#### Ethical approval

The MoBa study is conducted according to the Declaration of Helsinki for medical research involving human subjects. The establishment of MoBa and initial data collection was based on a license from the Norwegian Data Protection Agency. It is now based on regulations related to the Norwegian Health Registry Act. Participants provided written informed consent before joining the cohort. This project was approved by the Regional Committee for Medical and Health Research Ethics of South/East Norway (reference: 2017/1362).

### Method details

#### Use of centrally acting drugs

We used self-reported information on the use of centrally acting drugs from MoBa questionnaires in the 18^th^ and 30^th^ gestational weeks.^29^ Specifically, women were asked whether they had epilepsy or depression, and if they answered yes, whether any medications were used to treat the condition(s). This project has no information on the specific drug(s) the woman used. Any reported use of medication(s) for epilepsy (yes/no) or depression (yes/no) were categorized and evaluated separately.

#### Birth weight

Data on offspring birth weight in grams was obtained from the Medical Birth Registry of Norway, a national health registry which contains information about all births in Norway since 1967.^30^

#### Covariates

Information on maternal age at delivery (continuous), parity (1, 2, 3, ≥ 4) and sex of the offspring (female/male) was obtained from the birth registry. Furthermore, information on maternal years of education (continuous), pre-pregnancy body mass index (continuous), having ever smoked (yes/no), and self-reported depression or epilepsy was gathered in the questionnaire administered at gestational week 18.

#### Genetic variants related to placental transporters and genetic scores

We performed a systematic review in PubMed, Web of Science, pharmacogenetic websites (https://www.pharmgkb.org) and GWAS catalog (https://www.ebi.ac.uk/gwas/) to identify gene variants on the adenosine triphosphate-binding cassette superfamily of placental efflux transporters (MDR1-*ABCB1*, MRP1-*ABCC1,* MRP2-*ABCC2*, and BCRP-*ABCG2*). All databases were searched using the Boolean method with the following terms (1 AND 2 AND 3): 1) “antiseizure” OR “antiepilepsy” OR “anticonvulsants” OR “antidepressant” OR “benzodiazepines”; 2) “polymorphism” OR “genetic polymorphism” OR “genetic variant” OR “pharmacogenetics”; 3) “ATP binding cassette” or “*ABCB1*” OR “*ABCC1*” OR “*ABCC2*” OR “*ABCG2*” OR “*ABCC5*” OR “*ABCC3*”. In addition, the reference and discussions of all pooled articles were carefully scanned for additional publications. We excluded studies: 1) not performed in humans; 2) not published in English; 3) not published in scientific journals; and 4) focused on other drug groups. We followed the recommendations of the Preferred Reporting Items for Systematic reviews and Meta-Analyses statement (Table S1).^31^

After the systematic review, we only conserved genetic variants that were not in linkage disequilibrium according to linkage disequilibrium block analysis in the 1000 Genomes CEU and GBR populations (given the MoBa participants passing the post-imputation quality control cluster with these populations; *R*^*2*^ < 0.8) and with a minor allele frequency > 1% (Tables S2–S4).^32^ Genetic scores were calculated as the sum of the number of risk alleles for all genetic variants related to each transporter in a participant. The genetic score was represented in the results as a genetic score if the number of score values were ≤5. Otherwise, we represented them in quartiles. We considered a risk allele to be the allele associated with any of these theoretical situations potentially related to a decreased function of the transporter: declined expression of the transporter, non-resistance to the treatment, higher concentrations of the drug, or more adverse effects. Information related to the studies is available in Tables S2 and S3.

### Quantification and statistical analysis

#### Statistical analyses

Normally distributed continuous variables were described by means and standard deviations, nonnormally distributed continuous variables by medians and 25^th^-75^th^ percentiles, and categorical variables by proportions.

We first assessed the relationship between maternal use of centrally acting drugs during pregnancy and birth weight using multivariable linear regressions adjusted for offspring sex and maternal factors (age at delivery, years of education, pre-pregnancy body mass index, parity and having ever smoked). Clustered standard errors were computed to account for dependence among births to mothers contributing with more than one pregnancy in MoBa. To determine whether there were significant interactions between maternal prenatal use of centrally acting drugs and the genetic scores on the offspring birth weight, we applied likelihood ratio tests between nested linear regression models with and without an interaction product-term of “exposure group × genetic score”. The nested models were further adjusted for the first twenty ancestry-informative genetic principal components and genotyping batch. We considered any interaction with a *p*-value for the overall interaction test < 0.05 and a qualitative differential association between medication use and offspring birth weight in the two extreme categories according to the genetic score (a difference in the direction of the association estimators or a non-total overlap between the confidence intervals of the lowest and the highest scores). Whether an interaction was found, we further explored interactions with the individual variants in the genetic score as sensitivity analyses using the same strategy (as these were exploratory sensitivity analyses, we here considered any interaction with a *p*-value < 0.1).

Statistical analyses were performed in R Software, version 4.1.0.

## Supplemental information

Document S1. Figures S1‒S5 and Tables S1‒S4
