# Supplementary material for: Placental efflux transporters and antiseizure or antidepressant medication use impact birth weight in MoBa cohort
Source: iScience. 2024 Feb 20;27(3):109285. doi: 10.1016/j.isci.2024.109285 (PMC10918264; doi:10.1016/j.isci.2024.109285)
Supplement: Document S1. Figures S1‒S5 and Tables S1‒S4 [file mmc1.pdf]

iScience, Volume *27*

Supplemental information

Placental efflux transporters and antiseizure or antidepressant medication use impact birth weight in MoBa cohort

Marta H. Hernandez, Jacqueline M. Cohen, Karoline H. Skåra, Thea K. Grindstad, Yunsung Lee, Per Magnus, Pål R. Njølstad, Ole A. Andreassen, Elizabeth C. Corfield, Alexandra Havdahl, Espen Molden, Kari Furu, Maria C. Magnus, and Alvaro Hernaez

SUPPORTING INFORMATION

TABLE OF CONTENTS

SUPPLEMENTAL TABLES 2

Supplemental Table S1. STROBE checklist for cohort studies, related to Star Methods section Study participants. 2

Supplemental Table S2. Expected risk allele for birth weight in efflux transporters, related to Star Methods section Methods details. 3

Supplemental Table S3. The articles detail the development of the expected risk allele for the transporter score, related to Star Methods section Methods details. 4

Supplemental Table S4. The articles detail describing the findings from studies with negative results that were excluded, related to Star Methods section Methods details. 7

SUPPLEMENTAL FIGURES 10

Supplemental Figure S1. Flow chart of the systematic literature review of genetic variants on placental efflux transporters, related to Star Methods section Methods details. 10

Supplemental Figure S2. Differences in birth weight associated with pregnancy use of antiseizure drugs due to *ABCC2* individual genetic variants in the offspring, related to Figure 2. 11

Supplemental Figure S3. Differences in birth weight associated with pregnancy use of antiseizure drugs due to *ABCB1* individual genetic variants in the mother, related to Figure 3. 12

Supplemental Figure S4. Differences in birth weight in offspring exposed to prenatal antidepressant medication (vs. not exposed) in groups defined by offspring genetic scores for efflux transporters, related to figure 2. 13

Supplemental Figure S5. Differences in birth weight in offspring exposed to prenatal antidepressant medication (vs. not exposed) in groups defined by maternal genetic scores for efflux transporters, related to figure 3. 14

SUPPLEMENTAL REFERENCES 15

SUPPLEMENTAL TABLES

Supplemental Table S1. STROBE checklist for cohort studies, related to Star Methods section Study participants.

	Item No	Recommendation	Page No
**Title and abstract**			
Title and abstract	1	(*a*) Indicate the study's design with a commonly used term in the title or the abstract	1
		(*b*) Provide in the abstract an informative and balanced summary of what was done and what was found	3-4
**Introduction**			
Background/ rationale	2	Explain the scientific background and rationale for the investigation being reported	6-7
Objectives	3	State specific objectives, including any pre-specified hypotheses	6-7
**Methods**			
Study design	4	Present key elements of study design early in the paper	6
Setting	5	Describe the setting, locations, and relevant dates, including periods of recruitment, exposure, follow-up, and data collection	8
Participants	6	(*a*) Give the eligibility criteria, and the sources and methods of selection of participants. Describe methods of follow-up	8-9
		(*b*) For matched studies, give matching criteria and number of exposed and unexposed	-
Variables	7	Clearly define all outcomes, exposures, predictors, potential confounders, and effect modifiers. Give diagnostic criteria, if applicable	9-11
Data sources/ measurement	8	For each variable of interest, give sources of data and details of methods of assessment (measurement). Describe comparability of assessment methods if there is more than one group	8-11
Bias	9	Describe any efforts to address potential sources of bias	9-11
Study size	10	Explain how the study size was arrived at	Fig.1
Quantitative variables	11	Explain how quantitative variables were handled in the analyses. If applicable, describe which groupings were chosen and why	10
Statistical methods	12	(*a*) Describe all statistical methods, including those used to control for confounding	10-11
		(*b*) Describe any methods used to examine subgroups and interactions	10-11
		(*c*) Explain how missing data were addressed	-
		(*d*) If applicable, explain how loss to follow-up was addressed	-
		(*e*) Describe any sensitivity analyses	11
**Results**			
Participants	13*	(*a*) Report numbers of individuals at each stage of study—e.g. numbers potentially eligible, examined for eligibility, confirmed eligible, included in the study, completing follow-up, and analyzed	Fig. 1
		(*b*) Give reasons for non-participation at each stage	
		(*c*) Consider use of a flow diagram	
Descriptive data	14*	(*a*) Give characteristics of study participants (e.g. demographic, clinical, social) and information on exposures and potential confounders	12, Table 1
		(*b*) Indicate number of participants with missing data for each variable of interest	-
		(*c*) Summarize follow-up time (e.g., average and total amount)	-
Outcome data	15	Report numbers of outcome events or summary measures over time	12-13
Main results	16	(*a*) Give unadjusted estimates and, if applicable, confounder-adjusted estimates and their precision (e.g., 95% confidence interval). Make clear which confounders were adjusted for and why they were included	12-13 Figures 2,3
		(*b*) Report category boundaries when continuous variables were categorized	12-13 Figures 2,3
		(*c*) If relevant, consider translating estimates of relative risk into absolute risk for a meaningful time period	-
Other analyses	17	Report other analyses done—e.g. analyses of subgroups and interactions, and sensitivity analyses	13, Suppleme ntal figures 2-5
**Discussion**			
Key results	18	Summarize key results with reference to study objectives	14
Limitations	19	Discuss limitations of the study, taking into account sources of potential bias or imprecision. Discuss both direction and magnitude of any potential bias	15
Interpretation	20	Give a cautious overall interpretation of results considering objectives, limitations, multiplicity of analyses, results from similar studies, and other relevant evidence	14-15
Generalizability	21	Discuss the generalizability (external validity) of the study results	15-16
**Other information**			
Funding	22	Give the source of funding and the role of the funders for the present study and, if applicable, for the original study on which the present article is based	19

Supplemental Table S2. Expected risk allele for birth weight in efflux transporters, related to Star Methods section Methods details.

Gene	Polymorphism	Chromosome	Position	Expected risk allele for birth weight	Expected risk allele frequency in Europeans	References	Conserved after linkage disequilibrium block analysis
ABCB1	rs1045642	7	87138645	G	0.42	(1-9)	Yes
ABCB1	rs28401781	7	87148328	T	0.12	(10)	No
ABCB1	rs2235067	7	87149922	T	0.13	(11)	No
ABCB1	rs4148740	7	87152103	G	0.13	(12)	No
ABCB1	rs10280101	7	87153585	C	0.13	(11)	No
ABCB1	rs7787082	7	87157051	A	0.18	(11,13)	No
ABCB1	rs2032583	7	87160561	G	0.13	(11, 14-17)	No
ABCB1	rs2032582	7	87160618	T/A	A: 0.41<br>T: 0.02	(11, 15, 18-25)	No
ABCB1	rs4148739	7	87161049	C	0.13	(10-12)	No
ABCB1	rs11983225	7	87161520	C	0.13	(11)	No
ABCB1	rs10248420	7	87164986	G	0.18	(11, 26)	Yes
ABCB1	rs2235040	7	87165750	T	0.13	(11,15,16)	No
ABCB1	rs12720067	7	87169356	T	0.13	(11)	No
ABCB1	rs2032588	7	87179443	G	0.94	(27)	No, SNP not in MoBa
ABCB1	rs1128503	7	87179601	G	0.58	(5,12,22)	Yes
ABCB1	rs2235015	7	87199564	A	0.21	(11,17)	Yes
ABCB1	rs9282564	7	87229440	G	0.10	(28)	Yes
ABCB1	rs10245483	7	89637608	G	0.51	(29)	Yes
ABCC1	rs875740	16	16123048	C	0.35	(30)	Yes
ABCC1	rs212090	16	16236004	A	0.46	(31,32)	Yes
ABCC2	rs717620	10	101542578	C	0.79	(33-36)	Yes
ABCC2	rs4148386	10	101548468	G	0.44	(12)	No
ABCC2	rs2273697	10	101563815	G	0.80	(12, 37)	Yes
ABCC2	rs3740066	10	101604207	C	0.63	(12,33,38)	Yes
ABCG2	rs2231142	4	89052323	T	0.10	(39,40)	Yes
ABCG2	rs3114020	4	89083666	C	0.40	(40)	Yes

Supplemental Table S3. The articles detail the development of the expected risk allele for the transporter score, related to Star Methods section Methods details.

Gene	Polymorphism	Drug	Study participants	Effect		P-value	Expected risk allele in the publication	Risk allele for birth weight	Reference
ABCB1	rs1045642	All antiseizure drugs	114 drug-resistant epilepsy patients, 213 seizure-free patients and 287 controls (Taiwan)	Efficacy	The C allele and the CC genotype were associated with drug resistance relative to the T allele and the TT genotype.	0.001	T	G	1
ABCB1		All antiseizure drugs	746 patients with epilepsy and 179 controls (China)	Efficacy	The TT genotype was associated with drug resistance	0.0009	G		2
ABCB1		All antiseizure drugs	220 patients with epilepsy and 220 controls (India)	Efficacy	The TT genotype was associated with drug resistance relative to the CC genotype.	0.001	G		3
ABCB1		Carbamazepine	3,293 patients with epilepsy. Meta analysis	Pharmacokinetics/ Efficacy	The T allele was associated with lower concentrations, lower absorption, and resistance to the drug activity.	0.004	T		4
ABCB1		Carbamazepine	2,126 patients with epilepsy. Meta-analysis	Efficacy	The T allele was associated to decreased plasma concentrations of the drug.	<0.05	G		5
ABCB1		Agomelatine	28 healthy volunteers (Spain)	Pharmacokinetics	The TT genotype was associated with lower concentrations or lower absorption of the drug.	0.047	G		6
ABCB1		All antiseizure drugs	8,716 patients with epilepsy, 4,037 drug-resistant epilepsy patients and 4,679 drug-responsive patients. Meta analysis	Efficacy	The T allele was associated with higher risk of drug resistance in the overall population and in Caucasians.	0.006	G		7
ABCB1		Carbamazepine	In vitro	Impact of T allele on the sensitivity, intracellular accumulation, and transepithelial permeability of antiseizure drugs	The recombinant T allele_cells showed higher resistance to carbamazepine compared with C allele_cells in the cytotoxicity assay. The intracellular accumulation of carbamazepine was significantly decreased in cells transfecting with recombinant T allele when compared with recombinant C allele_cells.	0.01	G		8
ABCB1		Carbamazepine	210 patients with epilepsy (China)	Pharmacokinetics	The GG genotype was associated with increased concentrations of carbamazepine relative to the AG genotype.	0.001	G		9
ABCB1	rs10248420	All antidepressants	256 patients with epilepsy (Europe)	Efficacy	The G allele was associated with increased likelihood of remission when treated with amitriptyline, citalopram, paroxetine or venlafaxine relative to the A allele.	0.0095	G	G	11
ABCB1		Olanzapine	80 healthy volunteers (Spain)	Efficacy	The A allele was associated with decreased drug response compared to the G allele	0.047	G		26
ABCB1	rs1128503	All antiseizure drugs	90 patients with epilepsy (USA)	Pharmacokinetics	AA and AG genotypes were associated with increased clearance of carbamazepine relative to the GG genotype.	0.036	G	G	12
ABCB1		Lamotrigine	222 patients with epilepsy (Croatia)	Pharmacokinetics	The genotype CC was associated with higher lamotrigine concentrations compared to CT and TT genotypes.	0.021	G		22
ABCB1		Carbamazepine	2,126 patients with epilepsy. Meta-analysis	Efficacy	The CC genotype was associated with lower resistance to carbamazepine.	0.03	G		5
ABCB1	rs2235015	All antidepressants	339 patients with depression (European)	Efficacy	The A allele was associated with higher remission for P-glycoprotein antidepressants, citalopram, paroxetine, amitriptyline, and venlafaxine, relative to the C allele.	0.025	A	A	11

Gene	Polymorphism	Drug	Study participants	Effect		P-value	Expected risk allele in the publication	Risk allele for birth weight	Reference
ABCB1		All antidepressants	73 patients with depressive disorder (Germany)	Efficacy	The A allele was associated with increased likelihood of remission when treated with amitriptyline, citalopram, paroxetine or venlafaxine, relative to the C allele.	0.030	A		17
ABCB1	rs9282564	Paroxetine	71 psychiatric patients (Switzerland)	Efficacy	The G allele was associated with greater clinical response.	0.043	G	G	28
ABCB1	rs10245483	All antidepressants	683 patients with major depressive disorder (multisite)	Efficacy	The T allele was associated with less remission.	0.001	G	G	29
ABCC1	rs875740	All antiseizure drugs	199 patients from the Mayo Clinic Bipolar Disorder Biobank GWAS (USA)	Efficacy	The C allele was associated with better treatment response.	0.0089	C	C	30
ABCC1	rs212090	Clozapine	137 medicated patients (Australia)	Adverse effects	The A allele was associated with higher diastolic blood pressure (adverse effect)	0.01	A	A	31
ABCC1		All antidepressants	148 patients with major depressive disorder (Australia)	Efficacy	The A allele was included int the pharmacogenetic guide group and associated with greater remission	0.0001	A		32
ABCC2	rs717620	All antiseizure drugs	537 patients with epilepsy (217 drug resistant patients and 320 drug responders) (China)	Efficacy	The TT genotype was associated with drug resistance	0.001	C	C	33
ABCC2		All antiseizure drugs	4,300 patients with epilepsy (2,261 drug-resistant patients and 2,039 drug-responsive patients). Meta-analysis	Efficacy	The T allele was associated with drug resistance	0.0006	C		34
ABCC2		All antiseizure drugs	254 patients with epilepsy (104 drug-resistant and 150 drug-responsive) (China)	Efficacy	The TT genotype was associated with drug resistance, relative to the CC genotype	0.001	C		35
ABCC2		All antiseizure drugs	1,842 patients with epilepsy. Meta-analysis	Efficacy	The T allele was associated with drug resistance.	0.002	C		36
ABCC2	rs2273697	All antiseizure drugs	90 patients with epilepsy (USA)	Pharmacokinetics	AA and AG genotypes were associated with higher drug clearance relative to the GG genotype.	<0.05	G	G	12
ABCC2		All antiseizure drugs	453 patients with epilepsy (China)	Efficacy	The A allele A was associated with drug resistance.	0.001	G		37
ABCC2	rs3740066	All antiseizure drugs	90 patients with epilepsy (USA)	Pharmacokinetics	The G allele was associated with lower carbamazepine-10, 11-epoxide (metabolite): carbamazepine ratio.	0.008	C	C	12
ABCC2		All antiseizure drugs	537 patients with epilepsy (217 drug resistant patients and 320 drug responders) (China)	Efficacy	CT and TT genotypes were associated with drug resistance relative to CC genotype.	0.038	C		33
ABCC2		No drug	In vitro study	In vitro function of the transporter	The T allele was associated with a decreased function of the transporter.	0.017	C		38

Gene	Polymorphism	Drug	Study participants	Effect		P-value	Expected risk allele in the publication	Risk allele for birth weight	Reference
ABCG2	rs2231142	Lamotrigine	112 patients with epilepsy (China)	Pharmacokinetics	The TT genotype was associated with increased concentrations of lamotrigine relative to GG and GT genotypes.	0.015	T	T	39
ABCG2		Lamotrigine	140 patients with epilepsy (China)	Pharmacokinetics	CA and AA genotypes were associated with higher concentrations of lamotrigine relative to the CC genotype.	<0.05	T		40
ABCG2	rs3114020	Lamotrigine	140 patients with epilepsy (China)	Pharmacokinetics	CC and CT genotypes were associated with increased concentrations of lamotrigine relative to the TT genotype.	0.01	C	C	40

Supplemental Table S4. The articles detail describing the findings from studies with negative results that were excluded, related to Star Methods section Methods details.

Gene	Polymorphism	Drug	Effect	Study participants	Reference
ABCC2	rs717620, rs2273697 and rs3740066	Carbamazepine	Efficacy	229 patients on CBZ monotherapy	41
ABCC2	rs717620, rs2273697	Antiseizure	Efficacy	45 epilepsy patients drug responsive and 52 drug resistance	42
ABCC2	-	Carbamazepine	Pharmacokinetics	126 Mexican Mestizos (MM) with epilepsy.	43
ABCC2	rs717620, rs2273697 and rs3740066	Antiseizure	Efficacy	133 epilepsy patients drug resistant and 146 drug-responsive	44
ABCC2	rs717620, G-1774delG, rs3740066 and G2934A	Antiseizure	Efficacy	1302 drug-resistant patients and 1563 drug-sensitive controls were included	45
ABCC2	rs2273697 and rs3740066	Antiseizure	Efficacy	2056 epilepsy patients (Malaysia, Hong Kong and Japan)	46
ABCC2	rs3740066	Valproic acid	Efficacy	53 epilepsy patients (China).	47
ABCG2	rs2231137	Antiseizure	Efficacy	46 children with drug-resistant epilepsy and 47 healthy control subjects.	48
ABCC2, ABCC5 AND ABCG2	25 tagging SNPs	Antiseizure	Efficacy	262 epilepsy patients drug resistant and 328 drug responsive (China)	49
ABCB1	rs1128503, rs2032582 and rs1045642	Antiseizure	Efficacy	153 epileptic patients treated with antiseizure medication (Tunisia)	50
ABCB1	rs2032582	Risperidone	Efficacy	151 psychiatric patients (Caucasian)	51
ABCB1	-	Antidepressants	Efficacy	145 patients with Major Depression and 132 with bipolar disorder and 70 psychiatrically healthy (Korea)	52
ABCB1, ABCG2, AND ABCC2	ABCB1: rs1128503, rs2032582, rs1045642. ABCG2: 34G>A, 421C>A. ABCC2:rs2273697	Antiseizure	Efficacy	193 epilepsy patients drug responders and 198 non responders	53
ABCB1	rs1128503, rs2032582 and rs1045642	Antiseizure	Efficacy	Meta-analysis of 23 studies (7067 patients)	54
ABCB1	rs1128503, rs2032582, and rs1045642	Antiseizure	Efficacy	101 control subjects and 325 patients with epilepsy, of whom 94 were drug resistant and 231 drug responsive	55
ABCB1	rs1128503 and rs2032582	Valproic acid	Efficacy	249 epilepsy patients drug resistant and 256 drug responsive	56
ABCB1	rs1045642	Phenytoin	Toxicity	290 epilepsy patients and 14 controls	57
ABCB1	rs1045642 and rs2032582	Antiseizure	Overexpression	221 paediatric or adolescent epilepsy patients, 70 adult patients and 242 healthy volunteers (Caucasian)	58
ABCB1	rs1045642	Phenytoin	Efficacy	269 epilepsy patients	59
ABCB1	rs1128503, rs2032582 and rs1045642	Phenobarbitone, phenytoin, carbamazepine and valproate	Efficacy	392 consecutive epilepsy patients, out of which 228 had completed follow-up evaluation at 12 months.	60
ABCB1	rs1045642	Antiseizure	Efficacy	401 drug resistant epilepsy patients and 208 drug responsive	61
ABCB1	rs1128503, rs2032582, and rs1045642	Antiseizure	Efficacy	368 age- and sex-matched control children and 350 children epilepsy patients	62
ABCB1	rs1045642, rs3789243, rs1128503, rs2235046, rs1186746, and rs1186745	Antiseizure	Efficacy	242 epilepsy patients drug responsive and 198 were drug resistant	63

Gene	Polymorphism	Drug	Effect	Study participants	Reference
ABCB1	rs2032582 and rs10234411	Oxcarbazepine	Pharmacokinetics	40 patients were receiving oxcarbazepine monotherapy, 11 patients were placed in the oxcarbazepine bitherapy group combined with one enzyme-inducing anti-epileptic drugs, and 15 patients were placed in the oxcarbazepine bitherapy group combined with valproic acid.	64
ABCB1	rs1128503, rs2032582 and rs1045642	Carbamazepine (CBZ) or sodium valproate (VPA) monotherapy in epilepsy	Efficacy	685 epilepsy patients (China, India, and Malaysia)	65
ABCB1	rs1045642 and rs2032582	Antiseizure	Efficacy	69 patients with epilepsy drug resistant in and 83 drug responsive.	66
ABCB1	rs3789243, rs1128503, rs2032582, rs6949448 and rs1045642	Carbamazepine (CBZ) or sodium valproate (VPA) monotherapy	Efficacy	323 epilepsy patients drug resistant and 362 drug responsive (Malaysia)	67
ABCB1	rs1045642	Antidepressants	Efficacy	117 in-patients with a major depressive episode	68
ABCB1	rs1045642	Citalopram	Efficacy	54 patients with major depressive diagnose and 70 controls (Turkey)	69
ABCB1 AND ABCG2	rs1128503, rs2032582 and rs1045642, rs3213619, rs2214102, rs1202168 and rs1922242. ABCG2: rs2231142, rs72552713 and rs2231137	Antiseizure	Efficacy	259 epilepsy patients drug resistant, 201 patients drug responsive and 275 non-epilepsy control subjects.	70
ABCB1	rs1045642	Antiseizure	Efficacy	22 association studies including 3231 drug-resistant patients and 3524 drug-responsive patients or healthy controls (meta-analysis).	71
ABCB1	rs1045642, rs1128503, rs2032582	Sertraline	Pharmacokinetics	46 healthy volunteers (24 men and 22 women)	72
ABCB1 AND ABCC2	ABCB1: rs2032582 and rs1045642. ABCC2: rs717620 and rs2273697	Antiseizure	Efficacy	391 recruited subjects, 235 and 156 patients were classified into a drug responsive and resistant group (China).	73
ABCB1	rs1045642	Antidepressants (trazodone, sertraline, agomelatine and citalopram).	Pharmacokinetics	473 healthy volunteers	74
ABCB1, ABCG2	ABCB1: rs1128503, rs2032582 and rs1045642. ABCG2: rs2231142 and rs3114020	Lamotrigine	Pharmacokinetics	97 patients with epilepsy (Mexico)	75
ABCB1	rs2235040 and rs4148739	Antidepressants	Efficacy	152 patients were included from psychiatric departments (Rusia)	76
ABCB1	rs1045642	Antiseizure	Efficacy	85 patients	77
ABCB1	rs1045642 and rs2032582,	Antiseizure	Efficacy	39 patients with drug-resistant epilepsy and 92 controls.	78
ABCB1	rs1045642, rs2032582 and rs1128503	Antidepressants	Efficacy	112 major depressive disorder	79
ABCB1	rs1045642 and rs2032582	Paroxetine	Efficacy	127 patients with major depression	80
ABCB1	rs2032582 and rs10234411		Pharmacokinetics	88 patients with epilepsy (China)	81
ABCB1	rs1045642	Antiseizure	Efficacy	503 epilepsy patients with prospectively measured seizure and drug response outcomes	82
ABCB1	rs1045642	Phenytoin	TOXICITY	67 adult patients with epilepsy (Colombia)	83
ABCB1	rs1045642 and rs2032582	Antiseizure	Efficacy	2,134 patients	84

Gene	Polymorphism	Drug	Effect	Study participants	Reference
ABCB1	rs1128503, rs2032582, and rs1045642	Citalopram	Pharmacokinetics	1,953 subjects from the Sequenced Treatment Alternatives to Relieve Depression (STAR*D) trial into a discovery (n = 831) and validation set (n = 1,046)	85
ABCB1	rs10280101, rs7787082, rs2032583 and rs2235040	Duloxetine	Efficacy	250 outpatients with nonpsychotic major depressive disorder	86
ABCB1	rs1045642	Antiseizure	Efficacy	11 case-control studies involving 3,371 patients (1,646 patients with drug-resistant epilepsy and 1,725 controls) (Meta analysis)	87
ABCB1	rs1128503, rs2032582 and rs1045642	Phenytoin	Pharmacokinetics	64 patients with epilepsy currently treated with PHT in mono- (n=25) and polytherapy (n=39), (Mexico).	88
ABCB1	rs2032582	Antiseizure	Efficacy	15 studies (n=1773 drug-resistant, and n=2250 drug-responsive epilepsy cases) (Meta analysis)	89
ABCB1	rs1045642	Antiseizure	Efficacy	170 patients were classified as responders, with > or =12 months seizure freedom on current treatment. The remaining 230 patients were classified as nonresponses.	90
ABCB1	rs1045642	Antiseizure	Efficacy	106 patients with drug-resistant and 67 drug-responsive epilepsy, and 98 non-epileptic children (Poland)	91
ABCB1	rs1045642, rs2032583, rs2235040, and rs2235015	Antidepressants	Efficacy	292 patients with major depressive disorder	92
ABCB1	rs2032582	Paroxetine	Efficacy	61 patients	93
ABCB1	rs1045642	Antiseizure	Efficacy	134 drug-responsive epilepsy patients, 41 drug-resistant (n=41) epilepsy and 175 healthy controls	94
ABCB1	rs1128503, rs2032582 and rs1045642	Nortriptyline	Pharmacokinetics	67 healthy volunteers	95
ABCB1	rs1045642	Antiseizure	Efficacy	542 newly treated patients were enrolled (Australia, Scotland and Hong Kong)	96
ABCB1	rs1045642 and rs2032582	Venlafaxine	Efficacy	52 outpatients	97
ABCB1	rs1045642	Antiseizure	Efficacy	59 patients with drug-resistant epilepsy, 60 children with drug-responsive epilepsy and 76 healthy children were involved in this study.	98
ABCB1	rs1045642	Antiseizure	Efficacy	82 children and young adolescents up to 18 years old (Caucasian)	99
ABCB1	rs1045642	Antiseizure	Efficacy	8 studies, including 634 drug-resistant patients, 615 drug-responsive patients and 1,052 healthy controls (Meta-analysis)	100
ABCB1	rs1045642	Carbamazepine monotherapy	Efficacy	145 patients with epilepsy	101
ABCB1	rs1128503, rs1045642	Antiseizure	Efficacy	207 epilepsy patients (Korea)	102
ABCB1	rs1045642	Carbamazepine monotherapy	Efficacy	97 patients treated with CBZ monotherapy	103
ABCB1	rs1045642	Valproic acid	Pharmacokinetics	104 patients meeting DSM-IV criteria for bipolar disorder and 169 controls	104
ABCB1	-	Phenytoin	Pharmacokinetics	109 healthy voluntaries (Benin)	105
ABCB1	rs1045642, rs2032582 and rs1128503 (and tSNPs)	Antiseizure	Efficacy	149 unrelated Caucasian patients with epilepsy	106

SUPPLEMENTAL FIGURES

Supplemental Figure S1. Flow chart of the systematic literature review of genetic variants on placental efflux transporters, related to Star Methods section Methods details..

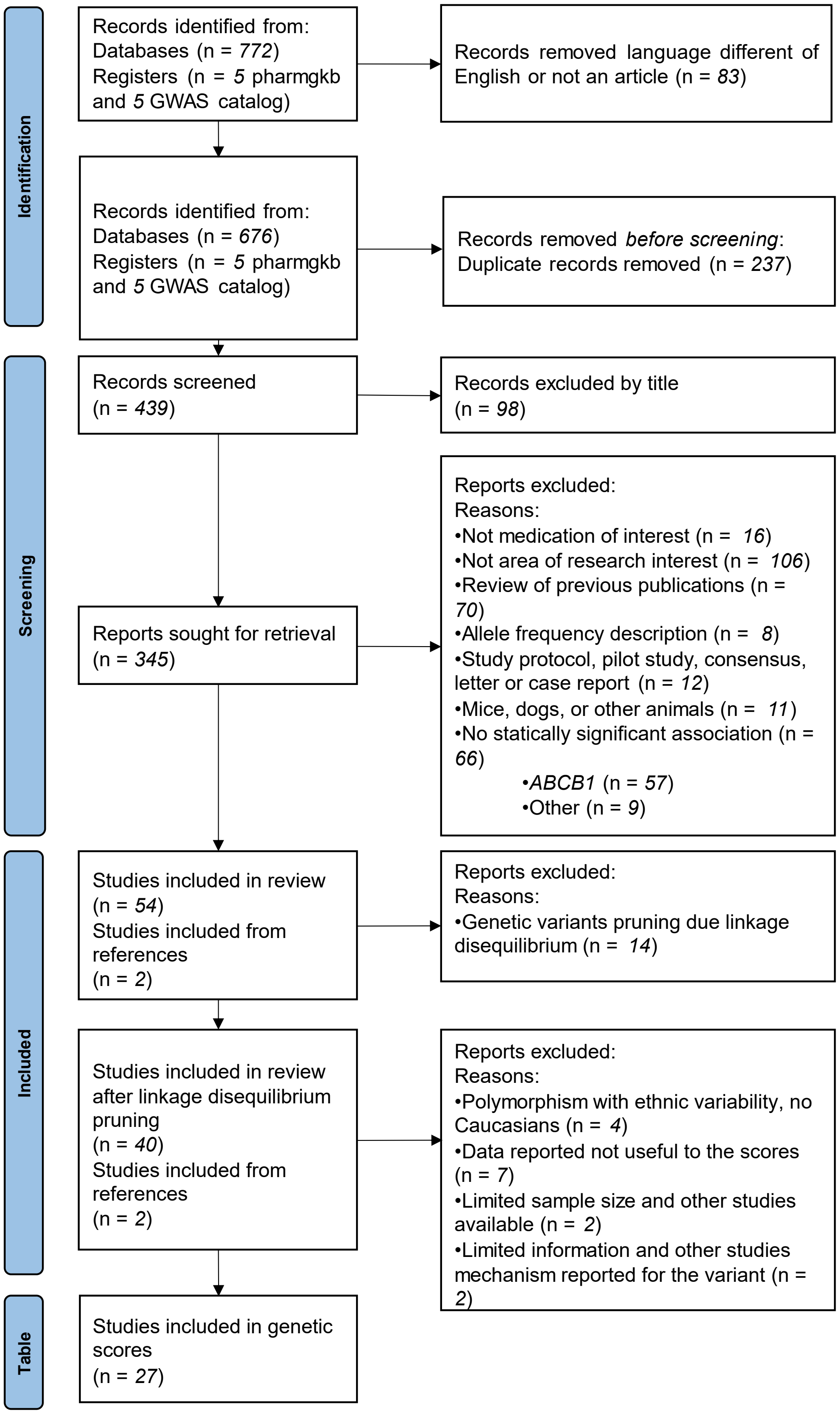

Supplemental Figure S2. Differences in birth weight associated with pregnancy use of antiseizure drugs due to *ABCC2* individual genetic variants in the offspring, related to Figure 2**.**

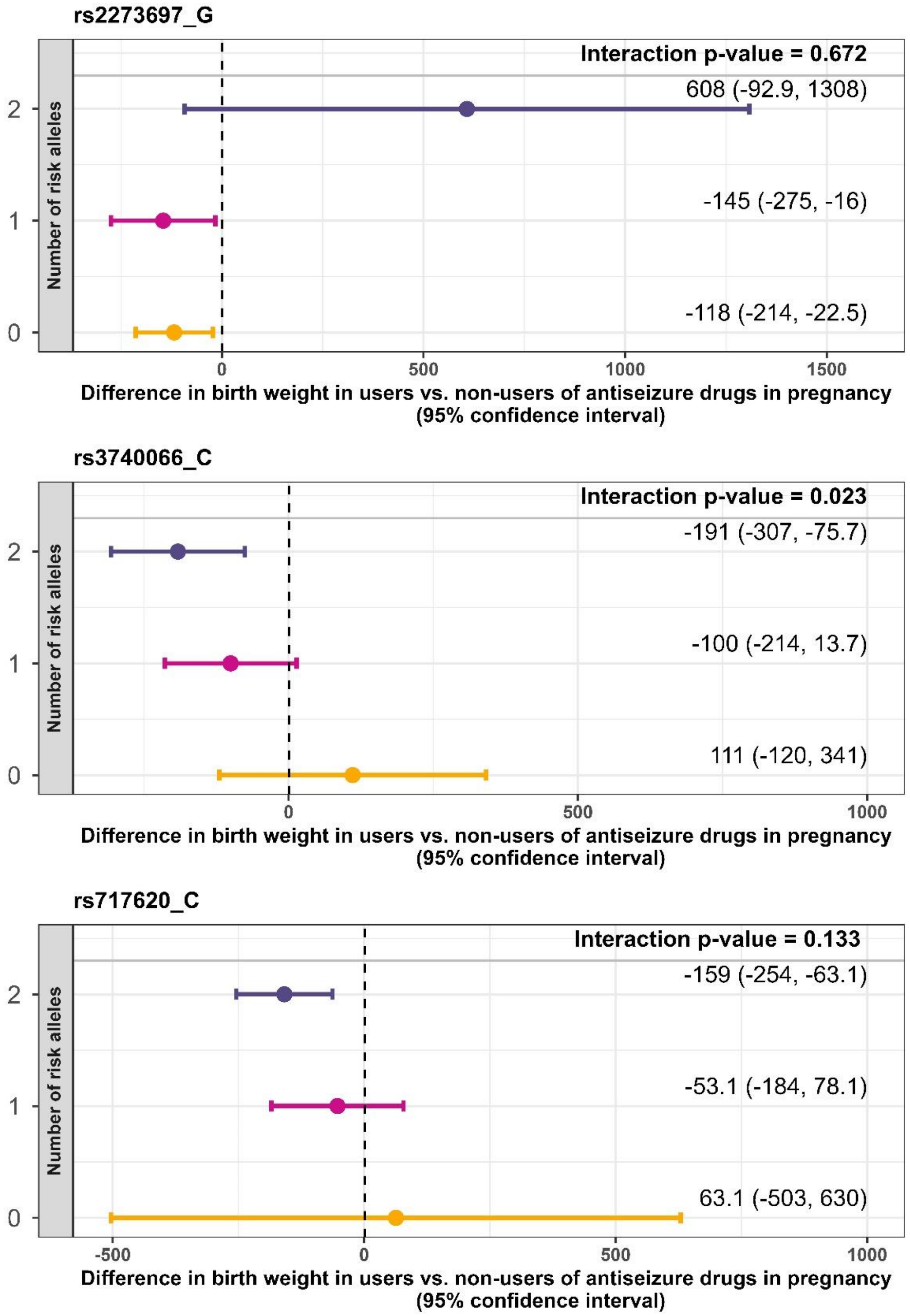

Supplemental Figure S3. Differences in birth weight associated with pregnancy use of antiseizure drugs due to *ABCB1* individual genetic variants in the mother, related to Figure 3.

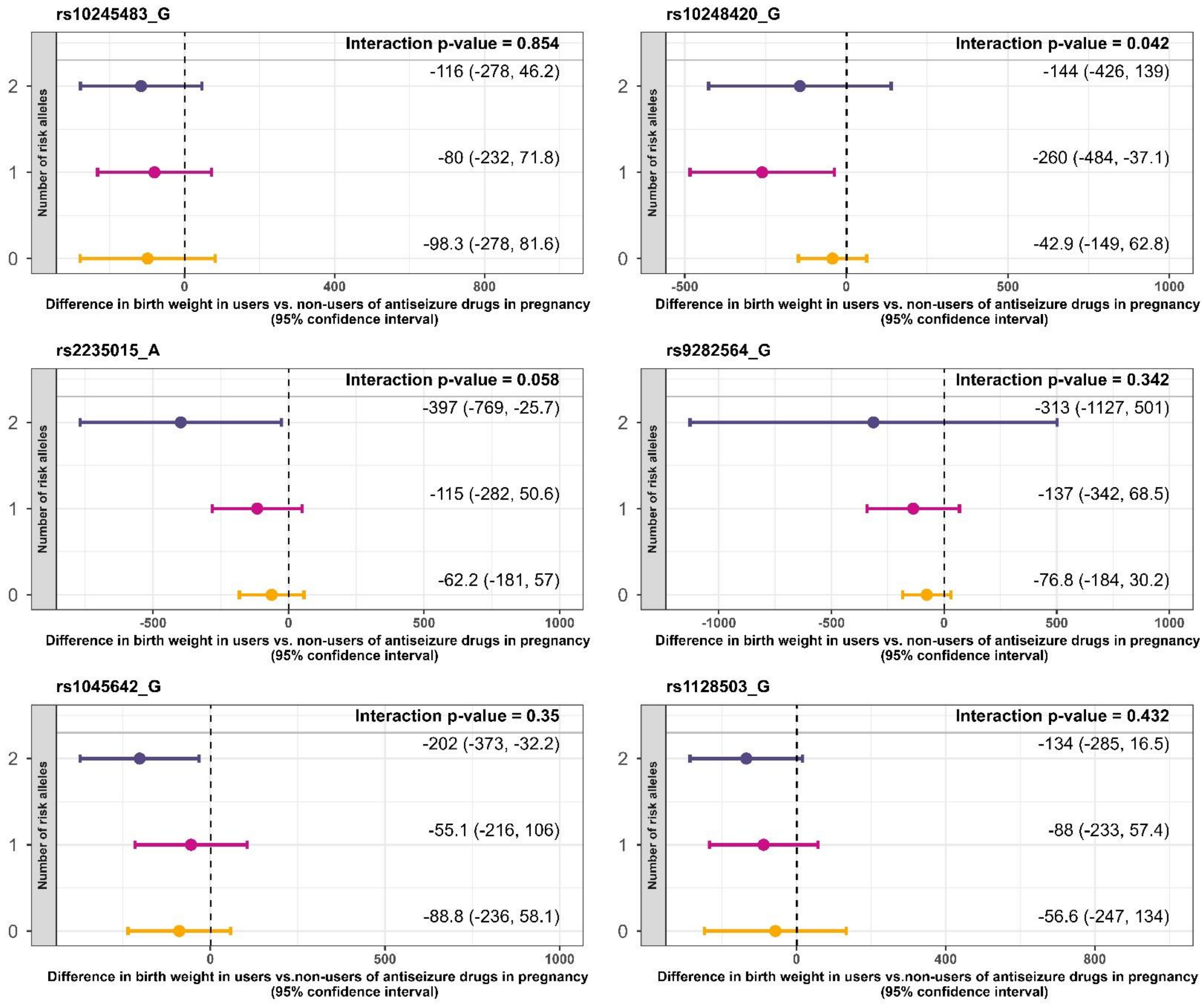

Supplemental Figure S4. Differences in birth weight in offspring exposed to prenatal antidepressant medication (vs. not exposed) in groups defined by offspring genetic scores for efflux transporters, related to figure 2.

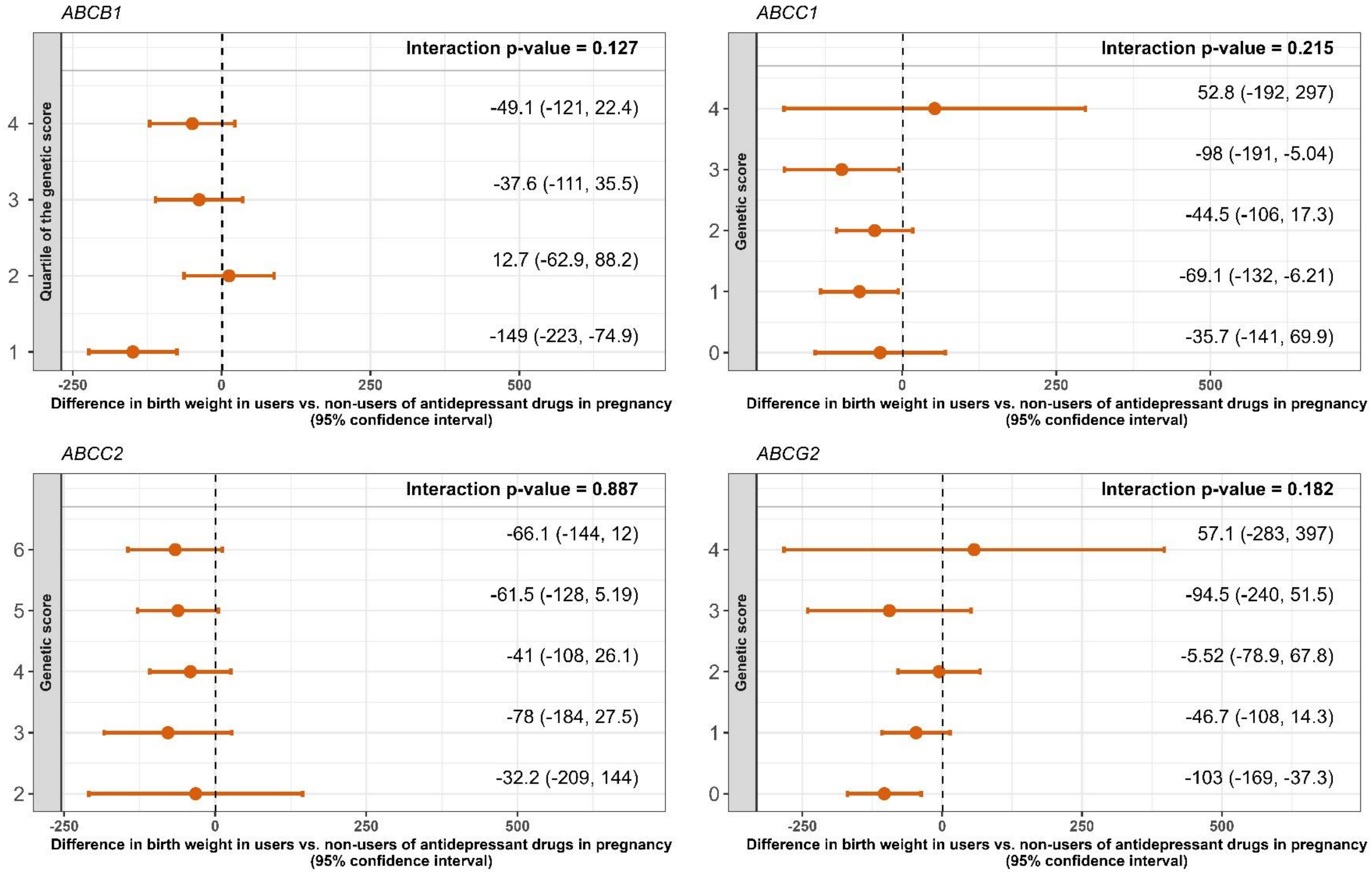

Supplemental Figure S5. Differences in birth weight in offspring exposed to prenatal antidepressant medication (vs. not exposed) in groups defined by maternal genetic scores for efflux transporters, related to figure 3.

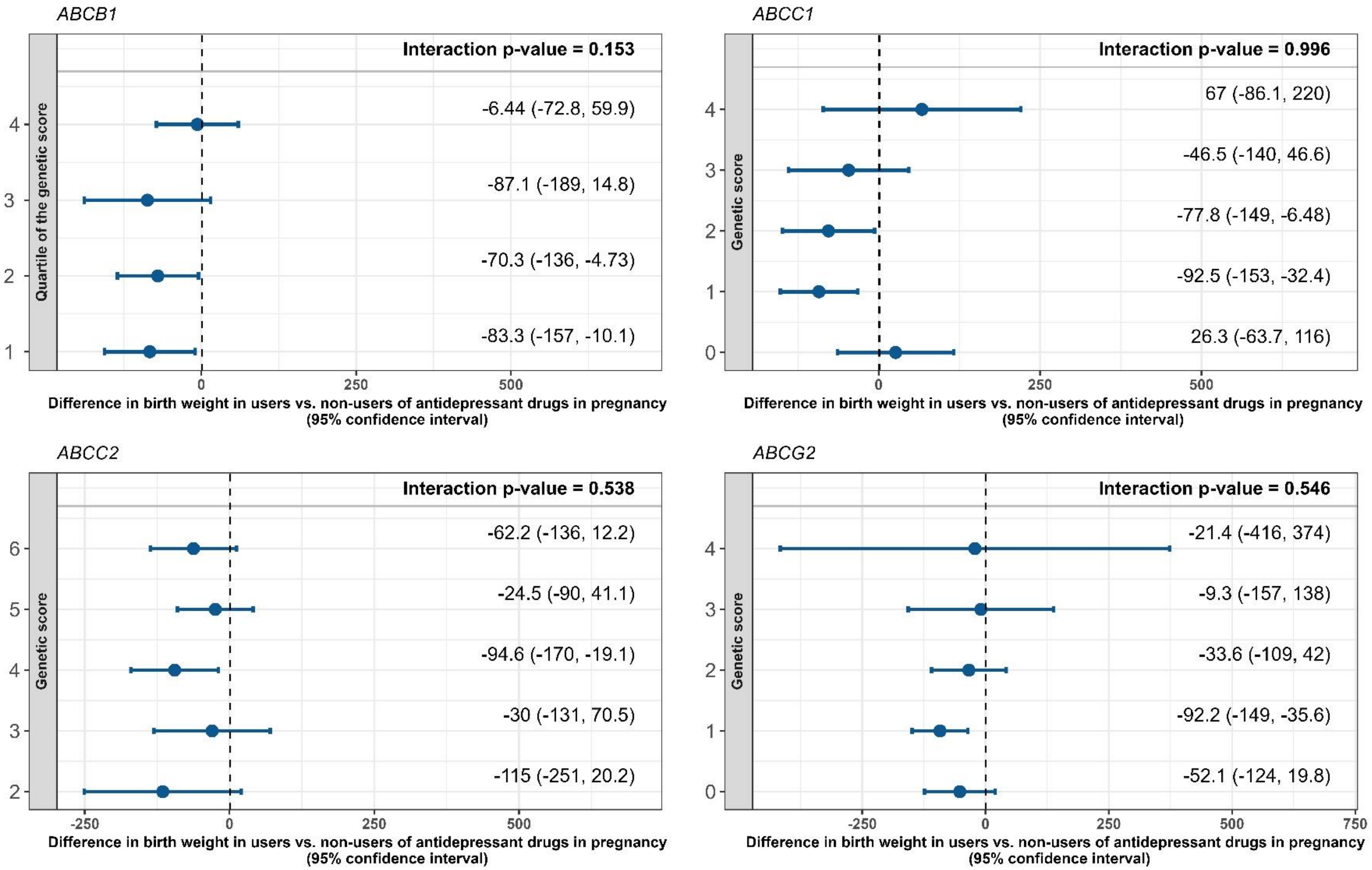

SUPPLEMENTAL REFERENCES

1. Hung, C.-C., Jen Tai, J., Kao, P.-J., Lin, M.-S., and Liou, H.-H. (2007). Association of polymorphisms inNR1I2andABCB1genes with epilepsy treatment responses. Pharmacogenomics 8, 1151–1158. 10.2217/14622416.8.9.1151.

2. Kwan, P., Baum, L., Wong, V., Ng, P.W., Lui, C.H.T., Sin, N.C., Hui, A.C.F., Yu, E., and Wong, L.K.S. (2007). Association between ABCB1 C3435T polymorphism and drug-resistant epilepsy in Han Chinese. Epilepsy Behav. 11, 112–117. 10.1016/j.yebeh.2007.04.013.

3. Shaheen, U., Prasad, D.K.V., Sharma, V., Suryaprabha, T., Ahuja, Y.R., Jyothy, A., and Munshi, A. (2014). Significance of MDR1 gene polymorphism C3435T in predicting drug response in epilepsy. Epilepsy Res. 108, 251–256. 10.1016/j.eplepsyres.2013.11.009.

4. Zhang, M.-L., Chen, X.-L., Bai, Z.-F., Zhao, X., Li, W.-X., Wang, X.-Y., Zhang, H., Chen, X.-F., Zhang, S.-Q., Tang, J.-F., et al. (2021). ABCB1 c.3435C > T and EPHX1 c.416A > G polymorphisms influence plasma carbamazepine concentration, metabolism, and pharmacoresistance in epileptic patients. Gene 805, 145907. 10.1016/j.gene.2021.145907.

5. Fan, Y.-X., Zhang, Z., Meng, J.-R., Yin, S.-J., Wang, P., Zhou, T., Huang, Y.-H., Meng, R., and He, G.-H. (2021). Association of ABCB1 polymorphisms with carbamazepine metabolism and resistance in epilepsy: A meta-analysis. Epilepsy Res. 177, 106785. 10.1016/j.eplepsyres.2021.106785.

6. Saiz-Rodríguez, M., Ochoa, D., Belmonte, C., Román, M., Vieira de Lara, D., Zubiaur, P., Koller, D., Mejía, G., and Abad-Santos, F. (2019). Polymorphisms in CYP1A2, CYP2C9 and ABCB1 affect agomelatine pharmacokinetics. J. Psychopharmacol. 33, 522–531. 10.1177/0269881119827959.

7. Li, M., Tan, J., Yang, X., Su, L., Xie, J., Liang, B., Long, J., Jiang, H., Wei, Q., Shen, T., et al. (2014). The ABCB1-C3435T polymorphism likely acts as a risk factor for resistance to antiepileptic drugs. Epilepsy Res. 108, 1052–1067. 10.1016/j.eplepsyres.2014.03.019.

8. Shen, X.-M., and Cheng, J. (2019). Effects ofMDR1(C3435T) polymorphism on resistance, uptake, and efflux to antiepileptic drugs. DNA Cell Biol. 38, 250–255. 10.1089/dna.2018.4553.

9. Zhu, X., Yun, W., Sun, X., Qiu, F., Zhao, L., and Guo, Y. (2014). Effects of major transporter and metabolizing enzyme gene polymorphisms on carbamazepine metabolism in Chinese patients with epilepsy. Pharmacogenomics 15, 1867–1879. 10.2217/pgs.14.142.

10. Huang, X., Yu, T., Li, X., Cao, Y., Li, X., Liu, B., Yang, F., Li, W., Zhao, X., Feng, G., et al. (2013). ABCB6, ABCB1 and ABCG1 genetic polymorphisms and antidepressant response of SSRIs in Chinese depressive patients. Pharmacogenomics 14, 1723–1730. 10.2217/pgs.13.151.

11. Uhr, M., Tontsch, A., Namendorf, C., Ripke, S., Lucae, S., Ising, M., Dose, T., Ebinger, M., Rosenhagen, M., Kohli, M., et al. (2008). Polymorphisms in the drug transporter gene ABCB1 predict antidepressant treatment response in depression. Neuron 57, 203–209. 10.1016/j.neuron.2007.11.017.

12. Puranik, Y.G., Birnbaum, A.K., Marino, S.E., Ahmed, G., Cloyd, J.C., Remmel, R.P., Leppik, I.E., and Lamba, J.K. (2013). Association of carbamazepine major

metabolism and transport pathway gene polymorphisms and pharmacokinetics in patients with epilepsy. Pharmacogenomics 14, 35–45. 10.2217/pgs.12.180.

13. Lee, S.-T., Ryu, S., Kim, S.-R., Kim, M.-J., Kim, S., Kim, J.-W., Lee, S.-Y., and Hong, K.S. (2012). Association study of 27 annotated genes for clozapine pharmacogenetics: Validation of preexisting studies and identification of a new candidate gene, ABCB1, for treatment response. J. Clin. Psychopharmacol. 32, 441–448. 10.1097/jcp.0b013e31825ac35c.

14. Breitenstein, B., Brückl, T.M., Ising, M., Müller-Myhsok, B., Holsboer, F., and Czamara, D. (2015). ABCB1gene variants and antidepressant treatment outcome: A meta-analysis. Am. J. Med. Genet. B Neuropsychiatr. Genet. 168, 274–283. 10.1002/ajmg.b.32309.

15. de Klerk, O.L., Nolte, I.M., Bet, P.M., Bosker, F.J., Snieder, H., den Boer, J.A., Bruggeman, R., Hoogendijk, W.J., and Penninx, B.W. (2013). ABCB1 gene variants influence tolerance to selective serotonin reuptake inhibitors in a large sample of Dutch cases with major depressive disorder. Pharmacogenomics J. 13, 349–353. 10.1038/tpj.2012.16.

16. Sarginson, J.E., Lazzeroni, L.C., Ryan, H.S., Ershoff, B.D., Schatzberg, A.F., and Murphy, G.M. (2010). ABCB1 (MDR1) polymorphisms and antidepressant response in geriatric depression. Pharmacogenet. Genomics 20, 467–475. 10.1097/fpc.0b013e32833b593a.

17. Breitenstein, B., Scheuer, S., Brückl, T.M., Meyer, J., Ising, M., Uhr, M., and Holsboer, F. (2016). Association of ABCB1 gene variants, plasma antidepressant concentration, and treatment response: Results from a randomized clinical study. J. Psychiatr. Res. 73, 86–95. 10.1016/j.jpsychires.2015.11.010.

18. Gassó, P., Rodríguez, N., Mas, S., Pagerols, M., Blázquez, A., Plana, M.T., Torra, M., Lázaro, L., and Lafuente, A. (2014). Effect of CYP2D6, CYP2C9 and ABCB1 genotypes on fluoxetine plasma concentrations and clinical improvement in children and adolescent patients. Pharmacogenomics J. 14, 457–462. 10.1038/tpj.2014.12.

19. Glauser, T.A., Holland, K., O'Brien, V.P., Keddache, M., Martin, L.J., Clark, P.O., Cnaan, A., Dlugos, D., Hirtz, D.G., Shinnar, S., et al. (2017). Pharmacogenetics of antiepileptic drug efficacy in childhood absence epilepsy: CAE Pharmacogenetics. Ann. Neurol. 81, 444–453. 10.1002/ana.24886.

20. Kato, M., Fukuda, T., Serretti, A., Wakeno, M., Okugawa, G., Ikenaga, Y., Hosoi, Y., Takekita, Y., Mandelli, L., Azuma, J., et al. (2008). ABCB1 (MDR1) gene polymorphisms are associated with the clinical response to paroxetine in patients with major depressive disorder. Progress in Neuro-Psychopharmacology and Biological Psychiatry 32, 398–404. 10.1016/j.pnpbp.2007.09.003.

21. Mittal, B., Kumari, R., Lakhan, R., Garg, R.K., Kalita, J., and Misra, U.K. (2011). Pharmacogenomic association study on the role of drug metabolizing, drug transporters and drug target gene polymorphisms in drug-resistant epilepsy in a north Indian population. Indian J. Hum. Genet. 17, 32. 10.4103/0971-6866.80357.

22. Lovrić, M., Božina, N., Hajnšek, S., Kuzman, M.R., Sporiš, D., Lalić, Z., Božina, T., and Granić, P. (2012). Association between lamotrigine concentrations and ABCB1 polymorphisms in patients with epilepsy. Ther. Drug Monit. 34, 518–525. 10.1097/ftd.0b013e31826517c6.

23. Rafaniello, C., Sessa, M., Bernardi, F.F., Pozzi, M., Cheli, S., Cattaneo, D., Baldelli, S., Molteni, M., Bernardini, R., Rossi, F., et al. (2018). The predictive value of ABCB1, ABCG2, CYP3A4/5 and CYP2D6 polymorphisms for risperidone and

aripiprazole plasma concentrations and the occurrence of adverse drug reactions. Pharmacogenomics J. 18, 422–430. 10.1038/tpj.2017.38.

24. Vijayan, N.N., Mathew, A., Balan, S., Natarajan, C., Nair, C.M., Allencherry, P.M., and Banerjee, M. (2012). Antipsychotic drug dosage and therapeutic response in schizophrenia is influenced by ABCB1 genotypes: a study from a south Indian perspective. Pharmacogenomics 13, 1119–1127. 10.2217/pgs.12.86.

25. Subenthiran, S., Abdullah, N.R., Muniandy, P.K., Joseph, J.P., Cheong, K.C., Ismail, Z., and Mohamed, Z. (2013). G2677T polymorphism can predict treatment outcome of Malaysians with complex partial seizures being treated with Carbamazepine. Genet. Mol. Res. 12, 5937–5944. 10.4238/2013.november.26.3.

26. Zubiaur, P., Soria-Chacartegui, P., Koller, D., Navares-Gómez, M., Ochoa, D., Almenara, S., Saiz-Rodríguez, M., Mejía-Abril, G., Villapalos-García, G., Román, M., et al. (2021). Impact of polymorphisms in transporter and metabolizing enzyme genes on olanzapine pharmacokinetics and safety in healthy volunteers. Biomed. Pharmacother. 133, 111087. 10.1016/j.biopha.2020.111087.

27. Bet, P.M., Verbeek, E.C., Milaneschi, Y., Straver, D.B.M., Uithuisje, T., Bevova, M.R., Hugtenburg, J.G., Heutink, P., Penninx, B.W.J.H., and Hoogendijk, W.J.G. (2016). A common polymorphism in the ABCB1 gene is associated with side effects of PGP-dependent antidepressants in a large naturalistic Dutch cohort. Pharmacogenomics J. 16, 202–208. 10.1038/tpj.2015.38.

28. Gex-Fabry, M., Eap, C.B., Oneda, B., Gervasoni, N., Aubry, J.-M., Bondolfi, G., and Bertschy, G. (2008). CYP2D6 and ABCB1 genetic variability: Influence on paroxetine plasma level and therapeutic response. Ther. Drug Monit. 30, 474–482. 10.1097/ftd.0b013e31817d6f5d.

29. Schatzberg, A.F., DeBattista, C., Lazzeroni, L.C., Etkin, A., Murphy, G.M., Jr, and Williams, L.M. (2015). ABCB1 genetic effects on antidepressant outcomes: A report from the iSPOT-D trial. Am. J. Psychiatry 172, 751–759. 10.1176/appi.ajp.2015.14050680.

30. Ho, A.M.-C., Coombes, B.J., Nguyen, T.T.L., Liu, D., McElroy, S.L., Singh, B., Nassan, M., Colby, C.L., Larrabee, B.R., Weinshilboum, R.M., et al. (2020). Mood-stabilizing antiepileptic treatment response in bipolar disorder: A genome-wide association study. Clin. Pharmacol. Ther. 108, 1233–1242. 10.1002/cpt.1982.

31. Piatkov, I., Caetano, D., Assur, Y., Lau, S.L., Jones, T., Boyages, S.C., and McLean, M. (2017). ABCB1 and ABCC1 single-nucleotide polymorphisms in patients treated with clozapine. Pharmgenomics. Pers. Med. 10, 235–242. 10.2147/pgpm.s142314.

32. Singh, A.B. (2015). Improved antidepressant remission in major depression via a pharmacokinetic pathway polygene pharmacogenetic report. Clin. Psychopharmacol. Neurosci. 13, 150–156. 10.9758/cpn.2015.13.2.150.

33. Qu, J., Zhou, B.-T., Yin, J.-Y., Xu, X.-J., Zhao, Y.-C., Lei, G.-H., Tang, Q., Zhou, H.-H., and Liu, Z.-Q. (2012). ABCC2 polymorphisms and haplotype are associated with drug resistance in Chinese epileptic patients: ABCC2 polymorphisms and epilepsy drug resistance. CNS Neurosci. Ther. 18, 647–651. 10.1111/j.1755-5949.2012.00336.x.

34. Qian, L., Fang, S., Yan, Y.-L., Zeng, S.-S., Xu, Z.-J., and Gong, Z.-C. (2017). The ABCC2 c.-24C > T polymorphism increases the risk of resistance to antiepileptic drugs: A meta-analysis. J. Clin. Neurosci. 37, 6–14. 10.1016/j.jocn.2016.10.014.

35. Xue, T., and Lu, Z.N. (2016). Association between the polymorphisms in the ATP-binding cassette genes ABCB1 and ABCC2 and the risk of drug-resistant epilepsy in a Chinese Han population. Genet. Mol. Res. 15. 10.4238/gmr15048752.

36. Grover, S., and Kukreti, R. (2013). A systematic review and meta-analysis of the role ofABCC2variants on drug response in patients with epilepsy. Epilepsia 54, 936–945. 10.1111/epi.12132.

37. Ma, C.-L., Wu, X.-Y., Zheng, J., Wu, Z.-Y., Hong, Z., and Zhong, M.-K. (2014). Association of SCN1A, SCN2A and ABCC2 gene polymorphisms with the response to antiepileptic drugs in Chinese Han patients with epilepsy. Pharmacogenomics 15, 1323–1336. 10.2217/pgs.14.89.

38. Laechelt, S., Turrini, E., Ruehmkorf, A., Siegmund, W., Cascorbi, I., and Haenisch, S. (2011). Impact of ABCC2 haplotypes on transcriptional and posttranscriptional gene regulation and function. Pharmacogenomics J. 11, 25–34. 10.1038/tpj.2010.20.

39. Shen, C.-H., Zhang, Y.-X., Lu, R.-Y., Jin, B., Wang, S., Liu, Z.-R., Tang, Y.-L., and Ding, M.-P. (2016). Specific OCT1 and ABCG2 polymorphisms are associated with Lamotrigine concentrations in Chinese patients with epilepsy. Epilepsy Res. 127, 186–190. 10.1016/j.eplepsyres.2016.09.004.

40. Zhou, Y., Wang, X., Li, H., Zhang, J., Chen, Z., Xie, W., Zhang, J., Li, J., Zhou, L., and Huang, M. (2015). Polymorphisms of ABCG2, ABCB1 and HNF4α are associated with Lamotrigine trough concentrations in epilepsy patients. Drug Metab. Pharmacokinet. 30, 282–287. 10.1016/j.dmpk.2015.05.002.

41. Rädisch, S., Dickens, D., Lang, T., Bonnett, L., Arlanov, R., Johnson, M.R., Schwab, M., Marson, A.G., and Pirmohamed, M. (2014). A comprehensive functional and clinical analysis of ABCC2 and its impact on treatment response to carbamazepine. Pharmacogenomics J. 14, 481–487. 10.1038/tpj.2014.5.

42. Sporis, D., Bozina, N., Basić, S., Lovrić, M., Babić, T., Susak, I., and Marković, I. (2013). Lack of association between polymorphism in ABCC2 gene and response to antiepileptic drug treatment in Croatian patients with epilepsy. Coll. Antropol. 37.

43. Fricke-Galindo, I., Jung-Cook, H., Martínez-Juárez, I.E., Monroy-Jaramillo, N., Ortega-Vázquez, A., Rojas-Tomé, I.S., Dorado, P., Peñas-Lledó, E., Llerena, A., and López-López, M. (2021). Relevance ofNR1I2variants on carbamazepine therapy in Mexican Mestizos with epilepsy at a tertiary-care hospital. Pharmacogenomics 22, 983–996. 10.2217/pgs-2021-0081.

44. Seo, T., Ishitsu, T., Oniki, K., Abe, T., Shuto, T., and Nakagawa, K. (2010). ABCC2 haplotype is not associated with drug-resistant epilepsy. J. Pharm. Pharmacol. 60, 631–635. 10.1211/jpp.60.5.0009.

45. Wang, Y., Tang, L., Pan, J., Li, J., Zhang, Q., and Chen, B. (2015). The recessive model of MRP2 G1249A polymorphism decrease the risk of drug-resistant in Asian Epilepsy: A systematic review and meta-analysis. Epilepsy Res. 112, 56–63. 10.1016/j.eplepsyres.2015.02.007.

46. Sha'ari, H.M., Haerian, B.S., Baum, L., Saruwatari, J., Tan, H.J., Rafia, M.H., Raymond, A.A., Kwan, P., Ishitsu, T., Nakagawa, K., et al. (2014). ABCC2 rs2273697 and rs3740066 polymorphisms and resistance to antiepileptic drugs in Asia Pacific epilepsy cohorts. Pharmacogenomics 15, 459–466. 10.2217/pgs.13.239.

47. Chen, J., Su, Q., Qin, J., Zhou, Y., Ruan, H., Chen, Z., Chen, Z., Li, H., Zhou, Y., Zhou, S., et al. (2019). Correlation of MCT1 and ABCC2 gene polymorphisms with valproic acid resistance in patients with epilepsy on valproic acid monotherapy. Drug Metab. Pharmacokinet. 34, 165–171. 10.1016/j.dmpk.2018.01.006.

48. Mousavi, S.F., Hasanpour, K., Nazarzadeh, M., Adli, A., Bazghandi, M.S., Asadi, A., Rad, A., and Gholami, O. (2022). ABCG2, SCN1A and CYP3A5 genes polymorphism and drug-resistant epilepsy in children: A case-control study. Seizure 97, 58–62. 10.1016/j.seizure.2022.03.009.

49. Kwan, P., Wong, V., Ng, P.W., Lui, C.H.T., Sin, N.C., Wong, K.S., and Baum, L. (2011). Gene-wide tagging study of the association between ABCC2, ABCC5 and ABCG2 genetic polymorphisms and multidrug resistance in epilepsy. Pharmacogenomics 12, 319–325. 10.2217/pgs.10.183.

50. Ajmi, M., Boujaafar, S., Zouari, N., Amor, D., Nasr, A., Rejeb, N.B., Amor, S.B., Omezzine, A., Benammou, S., and Bouslama, A. (2018). Association between ABCB1 polymorphisms and response to first-generation antiepileptic drugs in a Tunisian epileptic population. Int. J. Neurosci. 128, 705–714. 10.1080/00207454.2017.1412964.

51. Mas, S., Gassò, P., Álvarez, S., Parellada, E., Bernardo, M., and Lafuente, A. (2012). Intuitive pharmacogenetics: spontaneous risperidone dosage is related to CYP2D6, CYP3A5 and ABCB1 genotypes. Pharmacogenomics J. 12, 255–259. 10.1038/tpj.2010.91.

52. Crisafulli, C., Chiesa, A., Han, C., Lee, S.-J., Balzarro, B., Andrisano, C., Sidoti, A., Patkar, A.A., Pae, C.-U., and Serretti, A. (2013). Case-control association study of 36 single-nucleotide polymorphisms within 10 candidate genes for major depression and bipolar disorder. Psychiatry Res. 209, 121–123. 10.1016/j.psychres.2012.11.009.

53. Kim, D.W., Lee, S.K., Chu, K., Jang, I.-J., Yu, K.-S., Cho, J.-Y., and Kim, S.-J. (2009). Lack of association between ABCB1, ABCG2, and ABCC2 genetic polymorphisms and multidrug resistance in partial epilepsy. Epilepsy Res. 84, 86–90. 10.1016/j.eplepsyres.2008.12.001.

54. Haerian, B.S., Lim, K.S., Tan, C.T., Raymond, A.A., and Mohamed, Z. (2011). Association of ABCB1 gene polymorphisms and their haplotypes with response to antiepileptic drugs: a systematic review and meta-analysis. Pharmacogenomics 12, 713–725. 10.2217/pgs.10.212.

55. Lakhan, R., Misra, U.K., Kalita, J., Pradhan, S., Gogtay, N.J., Singh, M.K., and Mittal, B. (2009). No association of ABCB1 polymorphisms with drug-refractory epilepsy in a north Indian population. Epilepsy Behav. 14, 78–82. 10.1016/j.yebeh.2008.08.019.

56. Haerian, B.S., Lim, K.S., Tan, H.J., Mohamed, E.H.M., Tan, C.T., Raymond, A.A., Wong, C.P., Wong, S.W., Omar, H., Roslan, H., et al. (2011). Association between ABCB1 polymorphism and response to sodium valproate treatment in Malaysian epilepsy patients. Epileptic Disord. 13, 65–75. 10.1684/epd.2011.0419.

57. Hennessy, S., Leonard, C.E., Freeman, C.P., Metlay, J.P., Chu, X., Strom, B.L., and Bilker, W.B. (2009). CYP2C9, CYP2C19, and ABCB1 genotype and hospitalization for phenytoin toxicity. J. Clin. Pharmacol. 49, 1483–1487. 10.1177/0091270009343006.

58. Ufer, M., Mosyagin, I., Muhle, H., Jacobsen, T., Haenisch, S., Häsler, R., Faltraco, F., Remmler, C., von Spiczak, S., Kroemer, H.K., et al. (2009). Non-response to antiepileptic pharmacotherapy is associated with the ABCC2 −24C>T polymorphism in young and adult patients with epilepsy. Pharmacogenet. Genomics 19, 353–362. 10.1097/fpc.0b013e328329940b.

59. Hung, C.-C., Huang, H.-C., Gao, Y.-H., Chang, W.-L., Ho, J.-L., Chiou, M.-H., Hsieh, Y.-W., and Liou, H.-H. (2012). Effects of polymorphisms in six candidate genes on phenytoin maintenance therapy in Han Chinese patients. Pharmacogenomics 13, 1339–1349. 10.2217/pgs.12.117.

60. Grover, S., Bala, K., Sharma, S., Gourie-Devi, M., Baghel, R., Kaur, H., Gupta, M., Talwar, P., and Kukreti, R. (2010). Absence of a general association between ABCB1 genetic variants and response to antiepileptic drugs in epilepsy patients. Biochimie 92, 1207–1212. 10.1016/j.biochi.2010.04.008.

61. Tan, N.C.K., Heron, S.E., Scheffer, I.E., Pelekanos, J.T., McMahon, J.M., Vears, D.F., Mulley, J.C., and Berkovic, S.F. (2004). Failure to confirm association of a polymorphism in ABCB1 with multidrug-resistant epilepsy. Neurology 63, 1090–1092. 10.1212/01.wnl.0000137051.33486.c7.

62. Dong, L., Luo, R., Tong, Y., Cai, X., Mao, M., and Yu, D. (2011). Lack of association between ABCB1 gene polymorphisms and pharmacoresistant epilepsy: An analysis in a western Chinese pediatric population. Brain Res. 1391, 114–124. 10.1016/j.brainres.2011.03.028.

63. Shahwan, A., Murphy, K., Doherty, C., Cavalleri, G.L., Muckian, C., Dicker, P., McCarthy, M., Kinirons, P., Goldstein, D., and Delanty, N. (2007). The controversial association of ABCB1 polymorphisms in refractory epilepsy: An analysis of multiple SNPs in an Irish population. Epilepsy Res. 73, 192–198. 10.1016/j.eplepsyres.2006.10.004.

64. Wang, P., Yin, T., Ma, H.Y., Liu, D.Q., Sheng, Y.A., and Zhou, B.T. (2015). First analysis of the association between CYP3A4/5, ABCB1 genetic polymorphisms and oxcarbazepine metabolism and transport in Chinese epileptic patients with oxcarbazepine monotherapy and bitherapy. J. Pharm. Pharm. Sci. 18.

65. Haerian, B.S., Lim, K.S., Mohamed, E.H.M., Tan, H.J., Tan, C.T., Raymond, A.A., Wong, C.P., Wong, S.W., and Mohamed, Z. (2011). Lack of association of ABCB1 and PXR polymorphisms with response to treatment in epilepsy. Seizure 20, 387–394. 10.1016/j.seizure.2011.01.008.

66. Seven, M., Batar, B., Unal, S., Yesil, G., Yuksel, A., and Guven, M. (2014). The drug-transporter gene MDR1 C3435T and G2677T/A polymorphisms and the risk of multidrug-resistant epilepsy in Turkish children. Mol. Biol. Rep. 41, 331–336. 10.1007/s11033-013-2866-y.

67. Haerian, B.S., Lim, K.S., Mohamed, E.H.M., Tan, H.J., Tan, C.T., Raymond, A.A., Wong, C.P., Wong, S.W., and Mohamed, Z. (2011). Lack of association of ABCB1 haplotypes on five loci with response to treatment in epilepsy. Seizure 20, 546–553. 10.1016/j.seizure.2011.04.003.

68. Menu, P., Gressier, F., Verstuyft, C., Hardy, P., Becquemont, L., and Corruble, E. (2010). Antidepressants and ABCB1 gene C3435T functional polymorphism: A naturalistic study. Neuropsychobiology 62, 193–197. 10.1159/000319361.

69. Ozbey, G., Yucel, B., Taycan, S.E., Kan, D., Bodur, N.E., Arslan, T., Percin, F., Yuksel, N., Guzey, C., and Uluoglu, C. (2014). ABCB1 C3435T polymorphism is associated with susceptibility to major depression, but not with a clinical response to citalopram in a Turkish population. Pharmacol. Rep. 66, 235–238. 10.1016/j.pharep.2013.09.004.

70. Balan, S., Bharathan, S.P., Vellichiramal, N.N., Sathyan, S., Joseph, V., Radhakrishnan, K., and Banerjee, M. (2014). Genetic association analysis of ATP binding cassette protein family reveals a novel association of ABCB1 genetic variants with epilepsy risk, but not with drug-resistance. PLoS One 9, e89253. 10.1371/journal.pone.0089253.

71. Haerian, B.S., Roslan, H., Raymond, A.A., Tan, C.T., Lim, K.S., Zulkifli, S.Z., Mohamed, E.H.M., Tan, H.J., and Mohamed, Z. (2010). ABCB1 C3435T polymorphism

and the risk of resistance to antiepileptic drugs in epilepsy: A systematic review and meta-analysis. Seizure 19, 339–346. 10.1016/j.seizure.2010.05.004.

72. Saiz-Rodríguez, M., Belmonte, C., Román, M., Ochoa, D., Koller, D., Talegón, M., Ovejero-Benito, M.C., López-Rodríguez, R., Cabaleiro, T., and Abad-Santos, F. (2018). Effect of polymorphisms on the pharmacokinetics, pharmacodynamics and safety of sertraline in healthy volunteers. Basic Clin. Pharmacol. Toxicol. 122, 501–511. 10.1111/bcpt.12938.

73. Zhou, L., Cao, Y., Long, H., Long, L., Xu, L., Liu, Z., Zhang, Y., and Xiao, B. (2015). ABCB1, ABCC2, SCN1A, SCN2A, GABRA1 gene polymorphisms and drug resistant epilepsy in the Chinese Han population. Pharmazie 70.

74. Saiz-Rodríguez, M., Belmonte, C., Román, M., Ochoa, D., Jiang-Zheng, C., Koller, D., Mejía, G., Zubiaur, P., Wojnicz, A., and Abad-Santos, F. (2018). Effect of ABCB1 C3435T polymorphism on pharmacokinetics of antipsychotics and antidepressants. Basic Clin. Pharmacol. Toxicol. 123, 474–485. 10.1111/bcpt.13031.

75. Ortega-Vázquez, A., Fricke-Galindo, I., Dorado, P., Jung-Cook, H., Martínez-Juárez, I.E., Monroy-Jaramillo, N., Rojas-Tomé, I.S., Peñas-Lledó, E., Llerena, A., and López-López, M. (2020). Influence of genetic variants and antiepileptic drug co-treatment on lamotrigine plasma concentration in Mexican Mestizo patients with epilepsy. Pharmacogenomics J. 20, 845–856. 10.1038/s41397-020-0173-2.

76. Geers, L.M., Ochi, T., Vyalova, N.M., Losenkov, I.S., Paderina, D.Z., Pozhidaev, I.V., Simutkin, G.G., Bokhan, N.A., Wilffert, B., Touw, D.J., et al. (2022). Influence of eight ABCB1 polymorphisms on antidepressant response in a prospective cohort of treatment-free Russian patients with moderate or severe depression: An explorative psychopharmacological study with naturalistic design. Hum. Psychopharmacol. 37. 10.1002/hup.2826.

77. Emich-Widera, E., Likus, W., Kazek, B., Niemiec, P., Balcerzyk, A., Sieroń, A.L., and Żak, I. (2013). CYP3A5*3 and C3435T MDR1 polymorphisms in prognostication of drug-resistant epilepsy in children and adolescents. Biomed Res. Int. 2013, 1–7. 10.1155/2013/526837.

78. Alpman, A., Ozkinay, F., Tekgul, H., Gokben, S., Pehlivan, S., Schalling, M., and Ozkinay, C. (2010). Multidrug resistance 1 (MDR1) gene polymorphisms in childhood drug-resistant epilepsy. J. Child Neurol. 25, 1485–1490. 10.1177/0883073810368997.

79. Chang, H.H., Chou, C.-H., Yang, Y.K., Lee, I.H., and Chen, P.S. (2015). Association betweenABCB1polymorphisms and antidepressant treatment response in Taiwanese major depressive patients. Clin. Psychopharmacol. Neurosci. 13, 250–255. 10.9758/cpn.2015.13.3.250.

80. Mihaljevic Peles, A., Bozina, N., Sagud, M., Rojnic Kuzman, M., and Lovric, M. (2008). MDR1 gene polymorphism: therapeutic response to paroxetine among patients with major depression. Progress in Neuro-Psychopharmacology and Biological Psychiatry 32, 1439–1444. 10.1016/j.pnpbp.2008.03.018.

81. Wang, P., Yin, T., Ma, H.-Y., Liu, D.-Q., Sheng, Y.-H., Wang, C., and Zhou, B.-T. (2015). Effects of CYP3A4/5 and ABCB1 genetic polymorphisms on carbamazepine metabolism and transport in Chinese patients with epilepsy treated with carbamazepine in monotherapy and bitherapy. Epilepsy Res. 117, 52–57. 10.1016/j.eplepsyres.2015.09.001.

82. Leschziner, G., Jorgensen, A.L., Pirmohamed, M., Williamson, P.R., Marson, A.G., Coffey, A.J., Middleditch, C., Rogers, J., Bentley, D.R., Chadwick, D.W., et al. (2006). Clinical factors and ABCB1 polymorphisms in prediction of antiepileptic drug

response: a prospective cohort study. Lancet Neurol. 5, 668–676. 10.1016/s1474-4422(06)70500-2.

83. Calderon-Ospina, C.A., Galvez, J.M., López-Cabra, C., Morales, N., Restrepo, C.M., Rodríguez, J., Aristizábal-Gutiérrez, F.A., Velez-van-Meerbeke, A., Laissue, P., and Fonseca-Mendoza, D.J. (2020). Possible genetic determinants of response to phenytoin in a group of Colombian patients with epilepsy. Front. Pharmacol. 11. 10.3389/fphar.2020.00555.

84. Niitsu, T., Fabbri, C., Bentini, F., and Serretti, A. (2013). Pharmacogenetics in major depression: A comprehensive meta-analysis. Progress in Neuro-Psychopharmacology and Biological Psychiatry 45, 183–194. 10.1016/j.pnpbp.2013.05.011.

85. Peters, E.J., Slager, S.L., Kraft, J.B., Jenkins, G.D., Reinalda, M.S., McGrath, P.J., and Hamilton, S.P. (2008). Pharmacokinetic genes do not influence response or tolerance to citalopram in the STAR*D sample. PLoS One 3, e1872. 10.1371/journal.pone.0001872.

86. Perlis, R.H., Fijal, B., Dharia, S., Heinloth, A.N., and Houston, J.P. (2010). Failure to replicate genetic associations with antidepressant treatment response in duloxetine-treated patients. Biol. Psychiatry 67, 1110–1113. 10.1016/j.biopsych.2009.12.010.

87. Bournissen, F.G., Moretti, M.E., Juurlink, D.N., Koren, G., Walker, M., and Finkelstein, Y. (2009). Polymorphism of the MDR1/ABCB1 C3435T drug-transporter and resistance to anticonvulsant drugs: A meta-analysis. Epilepsia 50, 898–903. 10.1111/j.1528-1167.2008.01858.x.

88. Ortega-Vázquez, A., Dorado, P., Fricke-Galindo, I., Jung-Cook, H., Monroy-Jaramillo, N., Martínez-Juárez, I.E., Familiar-López, I., Peñas-Lledó, E., LLerena, A., and López-López, M. (2016). CYP2C9, CYP2C19, ABCB1 genetic polymorphisms and phenytoin plasma concentrations in Mexican-Mestizo patients with epilepsy. Pharmacogenomics J. 16, 286–292. 10.1038/tpj.2015.45.

89. Yu, L., Liao, W.-P., Yi, Y.-H., and Qiu, G. (2015). ABCB1 G2677T/A polymorphism is associated with the risk of drug-resistant epilepsy in Asians. Epilepsy Res. 115, 100–108. 10.1016/j.eplepsyres.2015.05.015.

90. Sills, G.J., Mohanraj, R., Butler, E., McCrindle, S., Collier, L., Wilson, E.A., and Brodie, M.J. (2005). Lack of association between the C3435T polymorphism in the human multidrug resistance (MDR1) gene and response to antiepileptic drug treatment. Epilepsia 46, 643–647. 10.1111/j.1528-1167.2005.46304.x.

91. Stasiołek, M., Romanowicz, H., Połatyńska, K., Chamielec, M., Skalski, D., Makowska, M., and Smolarz, B. (2016). Association between C3435T polymorphism of MDR1 gene and the incidence of drug-resistant epilepsy in the population of Polish children. Behav. Brain Funct. 12. 10.1186/s12993-016-0106-z.

92. Shan, X.-X., Qiu, Y., Xie, W.-W., Wu, R.-R., Yu, Y., Wu, H.-S., and Li, L.-H. (2019). ABCB1 gene is associated with clinical response to SNRIs in a local Chinese Han population. Front. Pharmacol. 10. 10.3389/fphar.2019.00761.

93. Vancova, Z., Cizmarikova, M., Dragasek, J., Zofcakova, S., Kolarcik, P., and Mojzis, J. (2018). Does G2677T polymorphism of the MDR1 gene make a difference in the therapeutic response to paroxetine in depressed patients in a Slovakian population? Med. Sci. Monit. 24, 3136–3145. 10.12659/msm.907434.

94. Manna, I., Gambardella, A., Labate, A., Mumoli, L., Ferlazzo, E., Pucci, F., Aguglia, U., and Quattrone, A. (2015). Polymorphism of the multidrug resistance 1 gene

MDR1/ABCB1 C3435T and response to antiepileptic drug treatment in temporal lobe epilepsy. Seizure 24, 124–126. 10.1016/j.seizure.2014.09.010.

95. Jensen, B.P., Roberts, R.L., Vyas, R., Bonke, G., Jardine, D.L., and Begg, E.J. (2012). Influence of ABCB1 (P-glycoprotein) haplotypes on nortriptyline pharmacokinetics and nortriptyline-induced postural hypotension in healthy volunteers. Br. J. Clin. Pharmacol. 73, 619–628. 10.1111/j.1365-2125.2011.04126.x.

96. Szoeke, C., Sills, G.J., Kwan, P., Petrovski, S., Newton, M., Hitiris, N., Baum, L., Berkovic, S.F., Brodie, M.J., Sheffield, L.J., et al. (2009). Multidrug-resistant genotype (ABCB1) and seizure recurrence in newly treated epilepsy: Data from international pharmacogenetic cohorts. Epilepsia 50, 1689–1696. 10.1111/j.1528-1167.2009.02059.x.

97. Ozbey, G., Celikel, F.C., Cumurcu, B.E., Kan, D., Yucel, B., Hasbek, E., Percin, F., Guzey, I.C., and Uluoglu, C. (2017). Influence ofABCB1polymorphisms and serum concentrations on venlafaxine response in patients with major depressive disorder. Nord. J. Psychiatry 71, 230–237. 10.1080/08039488.2016.1268203.

98. Saygi, S., Alehan, F., Atac, F.B., Erol, I., Verdi, H., and Erdem, R. (2014). Multidrug resistance 1 (MDR1) 3435C/T genotyping in childhood drug-resistant epilepsy. Brain Dev. 36, 137–142. 10.1016/j.braindev.2013.01.016.

99. Urbanek, K. (2014). Polymorphism of ABCB1/MDR1 C3435T in children and adolescents with partial epilepsy is due to different criteria for drug resistance – preliminary results. Med. Sci. Monit. 20, 1654–1661. 10.12659/msm.890633.

100. Sun, G., Sun, X., and Guan, L. (2014). Association of MDR1 gene C3435T polymorphism with childhood intractable epilepsy: a meta-analysis. J. Neural Transm. (Vienna) 121, 717–724. 10.1007/s00702-014-1169-3.

101. Daci, A., Beretta, G., Vllasaliu, D., Shala, A., Govori, V., Norata, G.D., and Krasniqi, S. (2015). Polymorphic variants of SCN1A and EPHX1 influence plasma carbamazepine concentration, metabolism and pharmacoresistance in a population of kosovar Albanian epileptic patients. PLoS One 10, e0142408. 10.1371/journal.pone.0142408.

102. Kim, Y.O., Kim, M.K., Woo, Y.J., Lee, M.C., Kim, J.H., Park, K.W., Kim, E.Y., Roh, Y.I., and Kim, C.J. (2006). Single nucleotide polymorphisms in the multidrug resistance 1 gene in Korean epileptics. Seizure 15, 67–72. 10.1016/j.seizure.2005.11.001.

103. Ozgon, G.O., Bebek, N., Gul, G., and Cine, N. (2008). Association of MDR1 (C3435T) polymorphism and resistance to carbamazepine in epileptic patients from Turkey. Eur. Neurol. 59, 67–70. 10.1159/000109264.

104. Turgut, G., Kurt, E., Sengul, C., Alatas, G., Kursunluoglu, R., Oral, T., Turgut, S., and Herken, H. (2009). Association of MDR1 C3435T polymorphism with bipolar disorder in patients treated with valproic acid. Mol. Biol. Rep. 36, 495–499. 10.1007/s11033-007-9206-z.

105. Allabi, A.C., Gala, J.-L., and Horsmans, Y. (2005). CYP2C9, CYP2C19, ABCB1 (MDR1) genetic polymorphisms and phenytoin metabolism in a Black Beninese population. Pharmacogenet. Genomics 15, 779–786. 10.1097/01.fpc.0000174787.92861.91.

106. Leschziner, G.D., Andrew, T., Leach, J.P., Chadwick, D., Coffey, A.J., Balding, D.J., Bentley, D.R., Pirmohamed, M., and Johnson, M.R. (2007). Common ABCB1 polymorphisms are not associated with multidrug resistance in epilepsy using a gene-

wide tagging approach. Pharmacogenet. Genomics 17, 217–220. 10.1097/01.fpc.0000230408.23146.b1.
